# Recent progress in the role of endogenous metal ions in doxorubicin-induced cardiotoxicity

**DOI:** 10.3389/fphar.2023.1292088

**Published:** 2023-12-08

**Authors:** Ni Zhou, Shanshan Wei, Taoli Sun, Suifen Xie, Jian Liu, Wenqun Li, Bikui Zhang

**Affiliations:** ^1^ Department of Pharmacy, The Second Xiangya Hospital, Central South University, Changsha, Hunan, China; ^2^ Institute of Clinical Pharmacy, Central South University, Changsha, Hunan, China; ^3^ School of Pharmacy, Central South University, Changsha, Hunan, China; ^4^ School of Pharmacy, Hunan University of Chinese Medicine, Changsha, Hunan, China

**Keywords:** doxorubicin, cardiotoxicity, iron, copper, zinc, calcium

## Abstract

Doxorubicin is a widely used anticancer drug in clinical practice for the treatment of various human tumors. However, its administration is associated with cardiotoxicity. Administration of doxorubicin with low side effects for cancer treatment and prevention are, accordingly, urgently required. The human body harbors various endogenous metal ions that exert substantial influences. Consequently, extensive research has been conducted over several decades to investigate the potential of targeting endogenous metal ions to mitigate doxorubicin’s side effects and impede tumor progression. In recent years, there has been a growing body of research indicating the potential efficacy of metal ion-associated therapeutic strategies in inhibiting doxorubicin-induced cardiotoxicity (DIC). These strategies offer a combination of favorable safety profiles and potential clinical utility. Alterations in intracellular levels of metal ions have been found to either facilitate or mitigate the development of DIC. For instance, ferroptosis, a cellular death mechanism, and metal ions such as copper, zinc, and calcium have been identified as significant contributors to DIC. This understanding can contribute to advancements in cancer treatment and provide valuable insights for mitigating the cardiotoxic effects of other therapeutic drugs. Furthermore, potential therapeutic strategies have been investigated to alleviate DIC in clinical settings. The ultimate goal is to improve the efficacy and safety of Dox and offer valuable insights for future research in this field.

## 1 Introduction

Doxorubicin (Dox), the first anthracycline used in clinical therapy, is a widely used class of antitumor antibiotics, commonly employed in the treatment of childhood leukemia, breast cancer, lymphoma, and sarcoma, among other conditions. Unfortunately, the clinical application of Dox in anticancer therapy is greatly limited by various systemic adverse reactions, which can lead to acute and chronic cardiomyopathy and, in severe cases, congestive heart failure, and the cardiotoxicity is considered irreversible (Ewer and Lippman, 2005). DIC is classified as acute and chronic, the clinical dose of Dox is 50–60 mg once every 3–4 weeks or 20–30 mg weekly for 3 weeks, repeated after 2–3 weeks of withdrawal, with a maximum cumulative dose of 550 mg/m^2^ (Li and Hill, 2014). Children’s dosage is about half that of an adult. Up to 25% of patients using Dox may experience DIC (Swain et al., 2003).

Dox inhibits the growth and proliferation of tumor cells by inhibiting topoisomerase II (Top2β), inserting into the double helix structure of DNA to unravel the double strand and inhibiting DNA and RNA synthesis (Kitakata et al., 2022). However, the exact mechanism of DIC is not fully deciphered, and it is only certain that oxidative stress and apoptosis play a key role in DIC. Numerous studies have identified various potential pathogenic mechanisms of DIC, including transcriptional dysregulation, calcium dysregulation, oxidative stress, DNA damage, nitric oxide release, inflammatory mediates, mitochondrial dysfunction, accumulation of iron in mitochondria, and dysregulation of autophagy due to the inhibition of Top2β (Renu et al., 2018). Therefore, it is important and advantageous to gain an understanding of the pathological factors that contribute to the onset or progression of DIC.

The human body needs trace elements such as iron, copper, zinc, calcium, manganese, etc., to maintain health and normal function of the system. These essential metal elements play a crucial role within cells, as many catalytic enzymes and proteins involved in biochemical processes heavily depend on them (Zoroddu et al., 2019). Disorders in iron metabolism can result in significant tissue degeneration, impairment of organ function, and potential development of cancer, ferroptosis activation may be a potential strategy to overcome mechanisms of resistance to conventional cancer therapy (Zhao et al., 2022). In mice mesothelioma models, reducing bioavailable copper through the use of D-pen, tetrathiomolybdate (TM), or trientine also reduced tumor growth and hindered tumor blood vessel formation (Ishida et al., 2013). Zinc can bind to Tau-specific amino acids to accelerate deposition, induce Tau abnormal phosphorylation, amplify its cytotoxicity, and aggravate cognitive impairment in Alzheimer’s patients (Hu et al., 2017). Calcium is chelated through the sarcoplasmic/endoplasmic Ca^2+^-ATPase (SERCa2a) after its release from the Ryanodine receptors (RyR2), and changes in these proteins in the circulation occur as the heart ages, leading to arrhythmias (Hamilton and Terentyev, 2019).

The disturbance of metal ion balance in cardiomyocyte of DIC patients is associated with oxidative stress and mitochondrial dysfunction, indicating the significant role of metal ions in the pathogenesis of DIC (Sheibani et al., 2022). In heart tissues affected by DIC, it is possible to observe alterations in the regulation of metal ions, including upregulation, downregulation, or dysregulation. In 2022, Abe et al. found that Dox accumulated in mitochondria by intercalating into mitochondrial DNA and decreased the abundance of 5′-aminolevulinate synthase 1, the rate-limiting enzyme in this process, thereby resulting in iron overload and ferroptosis in mitochondria in cultured cardiomyocytes (Abe et al., 2022). Clinical trials have been conducted in experimental animal models of DIC, using supplementation, chelation, or modulation of metal ions for treatment.

In the following section, we have reviewed the various roles of metal ions in the pathological mechanism of DIC and have discussed the roles of iron, copper, zinc, and calcium in triggering oxidative stress, mitochondrial dysfunction, DNA damage, among other effects.

## 2 Role and mechanism of ferroptosis in DIC

**FIGURE 1:**
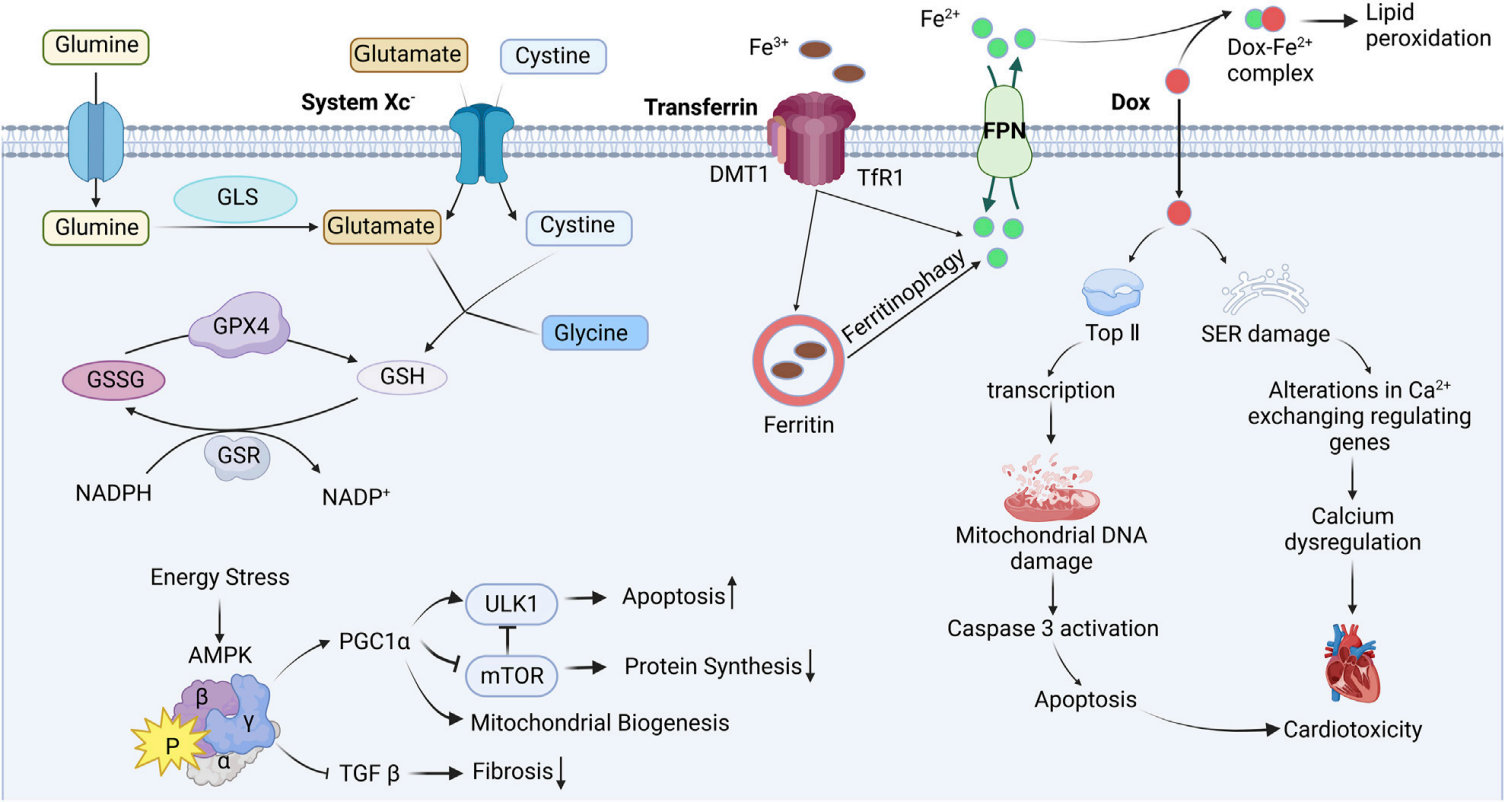
Mechanism of ferroptosis induced by Doxorubicin. Interference with iron metabolism after Dox administration, leads to DNA damage and endoplasmic reticulum stress, impairing mitochondrial function, ultimately resulting in cardiac toxicity. Blunt-ended lines indicate inhibition while arrows indicate promotion; DOX Doxorubicin; Top Ⅱ topoisomerase Ⅱ; SER sarcoplasmic (endoplasmic) reticulum; FPN ferroportin; DMT1 divalent metal-ion transporter 1; TfR1 transferrin receptor 1; GLS glutaminase; GSH glutathione; GSSG glutathione disulfide; GPX4 glutathione peroxidase 4; GSR glutathione reductase; NADPH nicotinamide adenine dinucleotide phosphate; AMPK AMP-activated protein kinase; PGC1α peroxisome proliferator–activated receptor-gamma coactivator 1α; TGF β transforming growth factor β; ULK1 Unc-51 like autophagy activating kinase; mTOR mammalian target of rapamycin. Image from biorender.

Ferroptosis is a newly discovered mode of regulatory cell death (RCD) by Stockwell et al., in 2002. Unlike other RCD modes. Ferroptosis does not show apoptotic features such as nuclear rupture, DNA ladder, and caspase-3 activation (Dolma et al., 2003), instead, it relies on Fe^2+^ and reactive oxygen species (ROS), along with mitochondrial dysfunction. This dysfunction is observed as mitochondrial outer membrane rupture, reduction or disappearance of mitochondrial ridges and shrinkage of the mitochondrial membrane (Friedmann Angeli et al., 2014). By focusing on the three primary features of ferroptosis, namely, dependence on iron, disrupted lipid metabolism, and excessive production of ROS (Jiang et al., 2021), new therapeutic targets can be pinpointed and more precise drugs can be developed. These are illustrated in Figure 1.

### 2.1 Iron overload

There are two main sources of dietary iron absorption: inorganic iron and heme iron (Uzel and Conrad, 1998). In a study conducted by Panjrath, G. S. et al., two groups of animals were fed either iron-rich food or ordinary food, and then treated with Dox and normal saline, respectively. The results showed that animals fed iron-rich food exhibited more severe DIC compared to those fed ordinary food. Additionally, feeding iron-rich foods alone did not cause cardiac damage, suggesting that iron accumulation and its bioavailability in the body may serve as crucial independent predictors of DIC sensitivity (Panjrath et al., 2007). The deposition of iron in cardiomyocyte can result in oxidative stress, mitochondrial damage and dysfunction, which further worsens DIC.

Disruption of the redox equilibrium is a core pathological mechanism in many heart diseases. The conversion of Fe^3+^ to Fe^2+^ is a reversible redox process, but electron transfer may result in the production of excessive ROS when iron is overloaded in cardiomyocyte. Normally, there is not enough free iron in the body to bind with Dox, the problem is that Dox carries a positive charge which enhances its affinity for iron and promotes the formation of the Dox-Fe complex. This complex alters the distribution and metabolism of iron and further combines with free oxygen in the body, resulting in cardiotoxicity (Gutteridge, 1984). Furthermore, iron and copper ions promote the binding of Dox to DNA, leading to increased cytotoxicity. A study demonstrated that 5-fluorouracil can upregulate iron levels in animals and cause iron accumulation in cell experiments, resulting in fluorescence quenching and increasing lipid peroxide levels (Li et al., 2022a). In addition, a study by He et al. revealed that pretreatment with Epigallocatechin-3-gallate can reduce iron deposition, inhibit oxidative stress by activating AMPKα2 (He et al., 2021). Recent studies have also shown that cardiac dysfunction caused by ferroptosis can be inhibited by iron complexing agents and antioxidants such as Ferrostatin-1 (Baba et al., 2018), Dexrazoxane (Weiss et al., 1999), Deferoxamine (Menon and Kim, 2022), Deferiprone (El-Ammar el et al., 2011), and Vitamin E (Berthiaume et al., 2005). These studies demonstrate that iron overload exacerbates Dox-induced oxidative stress and cardiomyocyte death. Interestingly, the use of antioxidants alone is insufficient to protect against DIC (Mukhopadhyay et al., 2009).

Cardiomyocyte contain a large number of mitochondria (Sheibani et al., 2022). However, mitochondria are also prone to iron overload, leading to lipid peroxidation on their membranes. Li et al. discovered that injection of endotoxin into mice increases the expression of nuclear receptor coactivator 4, facilitating the transfer of Fe^3+^ to mitochondria, leading to mitochondrial iron overload, triggering oxidative stress (Li et al., 2020b). On the other hand, Chang H.C. et al. suggested that overexpression of the mitochondrial iron efflux pump or the application of specifically targeted iron chelating agents within mitochondria could decrease mitochondrial iron levels (Chang et al., 2016). For instance, MitoTEMPO, a mitochondria-targeting antioxidant, can be easily integrated into the mitochondria (Dikalova et al., 2010). Experimental results have demonstrated that mitochondrial oxidative damage is the primary mechanism of heart injury caused by ferroptosis. Interestingly, the non-targeted antioxidant TEMPO does not exhibit any cardiac protective effects (Fang et al., 2019). Similarly, Ichikawa et al. confirmed that overexpression of the mitochondrial transporter ABCB8 promotes iron efflux, and prevents DIC *in vivo* and *in vitro* (Ichikawa et al., 2014). This suggests that reducing the iron levels in cardiomyocyte mitochondria can reverse the cardiotoxicity caused by Dox in cancer therapy.

### 2.2 Ferroptosis regulatory pathway

#### 2.2.1 Glutathione peroxidase 4 (GPX4)

The enzyme selenate Gpx4 plays a crucial role in regulating ferroptosis by minimizing lipid peroxidation. It accomplishes this by converting reduced glutathione (GSH) into glutathione disulfide (GSSG) and utilizing hydrogen ions to effectively diminish the presence of hydrogen peroxide and lipid peroxidation, subsequently reducing the accumulation of ROS (Forcina and Dixon, 2019). Several studies have reported that Dox can attenuate the levels of Gpx4. Protein arginine methyltransferase 4 can inhibit the Nrf2/Gpx4 to enhance ferroptosis, then mitigate DIC (Wang et al., 2022c). Moreover, the overexpression of Gpx4 or the introduction of ferrous ion chelators specifically targeting mitochondria have demonstrated the ability to mitigate DIC (Tadokoro et al., 2020).

#### 2.2.2 GSH/GSSG

GSH, a tripeptide comprising glutamine, cysteine and glycine, exists in cells in either its oxidized form, GSSG, or reduced form, GSH. GSH/GSSG is influenced by redox reaction, and maintaining an optimal ratio is vital for combating oxidative stress (Anderson, 1998). GSH catalytic detoxification of various electrophilic compounds and peroxides, facilitating the elimination of ROS through the, a process mediated by glutathione S-transferase and Gpx enzymes (Townsend et al., 2003). In a study conducted by Sun et al. observed that the protein expression of Gpx4 and SLC7A11, as well as the GSH/GSSG, were diminished upon Herceptin treatment (Sun et al., 2022). Furthermore, the intervention of Herceptin resulted in an increase in cellular and mitochondrial iron levels (Sun et al., 2022).

#### 2.2.3 System Xc-

System Xc-is a heterodimer reverse transport system for input cysteine and output glutamate, consisting of two subunits SLC3A2 and SLC7A11. Among them, SLC7A11 can promote cystine uptake and glutathione biosynthesis, maintain redox balance *in vivo*, and inhibit ferroptosis (Kim et al., 2020). In addition, the expression level of SLC7A11 is usually positively correlated with the activity of the reverse transporter (Lewerenz et al., 2013). Activation of transcription factor 3 (ATF3), a common stress receptor, inhibits SLC7A11 expression, thereby exacerbating elastin-induced lipid peroxidation and ferroptosis (Wang et al., 2020a). Repression of the SLC7A11/GSH/GPX4 axis triggers ferroptosis of vascular smooth muscle cells to promote vascular calcification under chronic kidney disease conditions (Ye et al., 2022).

#### 2.2.4 Nrf2-KEAP1

Nuclear factor erythroid 2-related factor 2 (Nrf2) possesses redox activity, when activated, iit enhances the overall detoxification and clearance of harmful substances in cells. Keap1 serves as an adapter protein for the CUL3-based E3 ubiquitin ligase and, under normal circumstances, binds to cytoplasmic Nrf2, promoting its ubiquitination and degradation. Hence, Keap1 functions as a negative inhibitor of Nrf2, a proposition that has also been experimentally supported through gene-deficient mice. Tertbutylhydroquinone (tBHQ) is an Nrf2 activator (Dinkova-Kostova and Talalay, 2008), the research demonstrates that tBHQ can induce Nrf2 overexpression, resulting in the upregulation of SLC7A11 expression in Hela or human corneal endothelial cells (Guha and Roy, 2021). In transgenic mice models, it has been proven that sequestration of Keap1 has been found to protect the heart from doxorubicin-induced ferroptosis (Hou et al., 2021). The ubiquitin E3 ligase TRIM21 interacts with p62 and ubiquitinates it, negatively regulating the p62-KEAP1-Nrf2 antioxidant pathway (Refaie et al., 2021).

#### 2.2.5 AMPK

Adenosine monophosphate-activated protein kinase (AMPK) is a macromolecular protein complex composed of three subunits: one catalytic site α and two regulatory sites β and γ. The AMPK pathway is closely associated with key mechanisms of DIC, including oxidative stress, mitochondrial damage, dysregulation of autophagy, increased apoptosis, and fibrosis. Dox inhibits the activation of other signals such as Akt and mitogen activated protein kinase (MAPK) through the AMPK pathway, leading to DNA damage. Activation of AMPK has several effects: firstly, it inhibits the autophagy activating protein ULK1, promoting autophagy (Kim et al., 2011); secondly, it inhibits the TGFβ pathway, which decreases myocardial fibrosis (Lin et al., 2015); thirdly, it inhibits the mTOR signal, reducing apoptosis (Ren et al., 2016). Various drugs can activate the AMPK signal, including metformin, statins, resveratrol, thiazolidinedione, AICAR (Acadesine), and specific AMPK agonists. These drugs are expected to have clinical applications in resisting DIC.

## 3 Promotion and treatment strategy of copper on DIC

Copper, an essential trace element in basic physiological activities of the human body, is involved in numerous key biological processes, including free radical scavenging, iron metabolism, connective tissue synthesis, immunity and cell signal transduction (Tainer et al., 1983; Banci and Bertini, 2013; Apresova et al., 2014; Bhuvanasundar et al., 2014). Additionally, several important “cuproproteins” in the human body, such as cytochrome C oxidase (COX), NADH dehydrogenase-2, Cu/Zn-superoxide dismutase (SOD1) and tyrosinase, rely on copper as a catalytic cofactor to fulfill their roles (Denoyer et al., 2015). Furthermore, the high redox activity of copper allows it to easily transition between the two valence states, and as a result, the generated electrons can promote the formation of ROS (Purchase, 2013).

**FIGURE 2:**
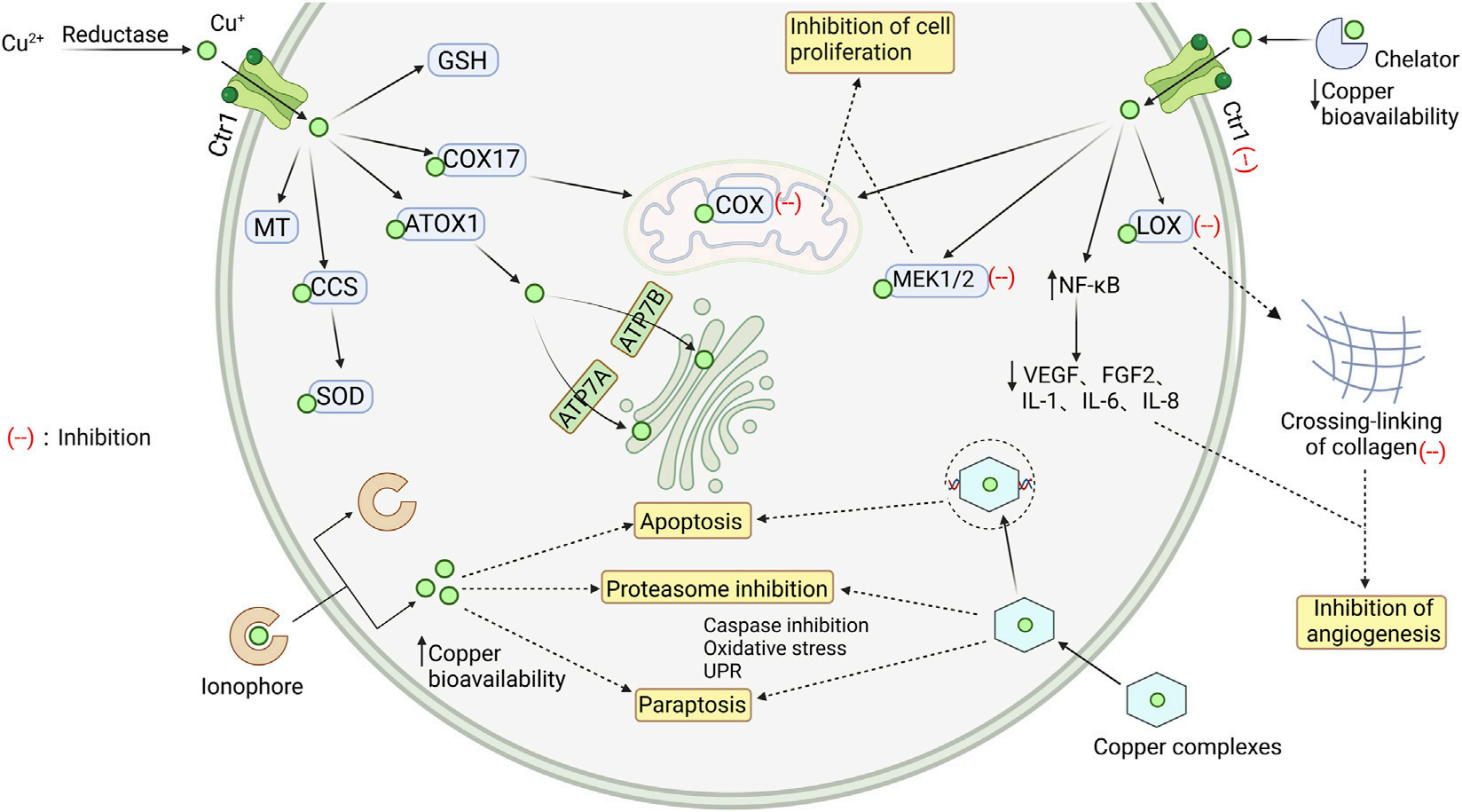
Intracellular transport of copper and the mechanism of chelating agents, ionophores, and complexes. The Cu^2+^ is reduced to Cu^+^, which then enters myocardial cells and binds to different proteins to maintain a steady state of copper ions in the cytoplasm. When there is an overload of copper ions, they can be removed by copper chelators, copper ionophores, and copper coordination compounds, which have a protective effect by clearing free copper ions. GSH glutathione; COX17 cytochrome c oxidase copper chaperone; ATOX1 antioxidant protein; CCS copper chaperone for SOD1; SOD Cu/Zn-superoxide dismutase; MT metallothionein; Ctr1 copper transporter 1; LOX recombinant lysyl oxidase; COX cytochrome c oxidase; MEK1/2 mitogen-activated protein kinase kinase; NF-κB nuclear factor kappa-B; VEGF vascular endothelial growth factor; FGF2 human basic fibroblast growth factor 2; ATP7A/B copper transporting ATPase A/B. Image from biorender.

In patients with osteosarcoma and leukemia, endogenous copper levels are higher compared to normal cells and tissues, and patients with more aggressive typically exhibit elevated serum copper levels (Fisher et al., 1976). Copper has been shown to promote angiogenesis, tumor growth, and metastasis (Gupte and Mumper, 2009). The body regulates copper concentrations through processes of absorption, excretion and bioavailability. Copper obtained from food sources (organic copper) undergoes processing by the liver, which controls its distribution in serum and tissues, excretes excess copper into bile (Angelova et al., 2011); on the other hand, copper (inorganic copper) present in drinking water or from copper supplements is absorbed by the intestinal mucosa and directly enters the free copper pool in the blood, bypassing the liver. This route likely allows copper to cross the blood-brain barrier (BBB) (Brewer, 2009). Based on Figure 2, three main approaches can be identified for addressing copper overload.

Copper deficiency may not necessarily worsen the cardiac toxicity induced by Dox (Fischer et al., 1993). It was found that copper-deficient rats may compensate for Dox-induced oxidative stress in the heart by improving the activity of Cu, Zn-superoxide dismutase and glutathione S-transfers (Fischer et al., 1992). However, Mizutani, H. et al. measured the levels of 8-oxo-7,8-dihydro-2′-deoxyguanosine (8-oxodG), a marker of DNA damage, and found that Dox can cause DNA damage in the presence of Cu^2+^ or cytochrome P450 reductase. Curiously, the extent of Cu^2+^-mediated DNA damage, including the formation of 8-oxodG, is far greater than the extent of DNA damage mediated by cytochrome P450 reductase (Mizutani et al., 2003). Therefore, people are striving to maintain stable levels of copper within cells when using Dox. This is currently being done through three approaches: chelation, ion carriers, and coordination compounds.

### 3.1 Copper chelation

The use of copper chelators to reduce the concentration and bioavailability of copper *in vivo* is an effective method. By forming complexes with copper, chelators help remove it from the body and inhibit the formation of cancer blood vessels, thereby preventing further progression of the disease.

Several common copper chelators, such as D-pen, trientine, ALXN 1840, or TM, have been shown to delay the spread of cancer by inhibiting angiogenesis in various animal models. This effect has been demonstrated in animal models of rat gliosarcoma (Brem et al., 1990), mice hepatocellular carcinoma (Yoshii et al., 2001), head and neck squamous cell carcinoma (Cox et al., 2001) and mesothelioma (Crowe et al., 2013). D-PEN, for example, can inhibit the activity of lysine oxidase and disrupt collagen crosslinking. Consequently, it affects the expression of endothelial growth factor, leading to the delayed progression of glioblastoma multiforme *in vivo* (Mammoto et al., 2013). Animal experiments have shown that treatment with trientine at doses ranging from approximately 34–68 mg/kg/day for 6–8 weeks resulted in increased cardiac output, increased left ventricular pressure, as well as improvements in left ventricular fractional shortening (LVFS) and left ventricular ejection fraction (LVEF). TM-induced copper deficiency inhibits angiogenesis by activating the transcription factor NF-κB, thereby reducing the secretion of angiogenic factors (VEGF, FGF2) and interleukins (IL-1a, IL-6, IL-8) (Pan et al., 2003). ALXN1840 acts by forming a tripartite complex with copper and albumin, which reduces the levels of non-ceruloplasmin-bound copper in the bloodstream. It specifically targets intracellular copper in the liver and increases the copper excretion through bile (Komatsu et al., 2000).

### 3.2 Copper ionophore

Copper ionophores are a class of fat-soluble molecules that reversibly bind copper ions and can induce apoptosis in copper-containing cells. Unlike copper chelators, which remove copper from the body, copper ionophores redistribute the distribution of copper ions within cells. By doing so, they exploit the cytotoxicity of copper to specifically target and eliminate cancer cells.

Three different compounds—Cu^2+^(gtsm) (a bis(thiosemicarbazone) analog), clioquinol (a hydroxyquinoline analog) and disulfiram (a dithiocarbamate analog)—have been studied for their ability to inhibit proteasomal chymotrypsin-like activity, thereby preventing the release of copper (Chen et al., 2007). These compounds have demonstrated anticancer effects both *in vitro* and in rodent models (Cater et al., 2013). Cu^2+^(gtsm), when used in combination with its ligands, exhibited enhanced anti-proliferative activity against tumor cells compared to the ligands alone (Anjum et al., 2019). It is worth noting that the growth of tumors in an anoxic microenvironment diminishes the anti-proliferative efficacy of most bis(thiosemicarbazone) compounds and their copper complexes, compared to an oxygen-rich environment (Anjum et al., 2019). Clioquinol and disulfiram have undergone extensive clinical trials, the results have shown that these compounds can indeed transport copper into human cells and exert selective cytotoxicity in tumor cells. However, the underlying reasons for this selectivity have not been identified (Johansson, 1992; Schimmer et al., 2012). Studies have demonstrated that the anticancer activity of clioquinol is intensified with higher extracellular copper levels, which can be counteracted by the formation of copper complexes with a chelating agent called TM (Cater and Haupt, 2011). Li et al.'s study showed that the disulfiram-copper complex exerts anti-tumor activity in nasopharyngeal carcinoma cells through the ROS/MAPK pathway and the p53-mediated ferroptosis pathway. Additionally, this complex can inhibit α-SMA expression, inactivate cancer-related fibroblasts (Li et al., 2020c).

### 3.3 Copper coordination compounds

The interest in finding alternative metal complexes for supplementary treatment has been growing, considering the widespread clinical use of platinum-based drugs such as cisplatin, carboplatin, and oxaliplatin. This is driven by the aim of reducing the side effects associated with platinum-containing chemotherapy (Marzano et al., 2009). Copper has been shown to have potential to enhance efficacy, reduce side effects, and bypass drug resistance (Marzano et al., 2009). A study conducted by Pivetta evaluated the combined effects of three copper coordination compounds containing one or two 1,10-phenanthroline molecules in combination with cisplatin. The results indicated a significant synergistic anti-tumor effect on cell proliferation. This combination therapy holds promise for enhancing the effectiveness of existing treatments (Pivetta et al., 2015). Additionally, the combination of copper and tin binary complexes has been investigated, demonstrating superior anticancer activity compared to monometallic complexes (Chauhan et al., 2007). CuSn2 has been particularly effective in inducing apoptotic cell death in various cancer cell lines *in vitro*. Notably, CuSn2 exhibits a significantly higher maximum tolerated dose than cisplatin while causing fewer toxic side effects. Interestingly, there is no evidence of liver, kidney, or brain toxicity associated with CuSn2 at equivalent doses (Zaidi et al., 2014).

In conclusion, the increasing research on copper complexes as complementary chemotherapy agents presents an encouraging avenue for cancer treatment. Novel copper complexes, such as the binuclear Cu^2+^ complex targeting DNA, combinations with existing drugs like cisplatin, and heterologous copper-tin complexes, hold promise for future cancer therapies. Further studies are warranted to fully explore the efficacy and safety profiles of these complexes and their potential for clinical translation.

## 4 Regulation of zinc and promoting effect on DIC and potential therapeutic direction

Zinc is the second most abundant metal element in the human body after iron. The majority of enzymatic reactions in the human body rely on zinc, making it indispensable for normal physiological functions and overall health. Zinc is found in various tissues and organs, but its content in the heart is minimal, accounting for 0.4% of the total zinc content (King et al., 2000). With age and the presence of certain pathological conditions, the level of zinc in the body gradually decreases, leading to an increased risk of complications (Bayır et al., 2013). Decreased zinc ion concentration in peripheral blood has been suggested as an independent risk factor for predicting coronary heart disease, especially in elderly patients, non-smokers, and postmenopausal women (Meng et al., 2021).

The [Zn^2+^]_i_ in Dox-treated H9c2 cells is significantly higher compared to the control group, indicating the presence of endoplasmic reticulum (ER) stress as evidenced by elevated levels of ER stress markers, such as GRP78 and CHOP/Gadd15 (Olgar et al., 2018). Interestingly, directly increasing [Zn^2+^]_i_ through a zinc ion carrier induced a significant increase in these markers, while directly inducing ER stress did not alter the levels of zinc ion transport proteins (Olgar et al., 2018). Disruption of Zn^2+^ homeostasis in cells can lead to mitochondrial and endoplasmic reticulum stress, causing disruption of normal ER/mitochondrial crosstalk and mitochondrial autophagy, resulting in metabolic dysfunction (Dabravolski et al., 2022). Therefore, controlling intracellular zinc levels through zinc ion transporters may be an important approach in the treatment of DIC.

### 4.1 Zinc ion transporters

**FIGURE 3:**
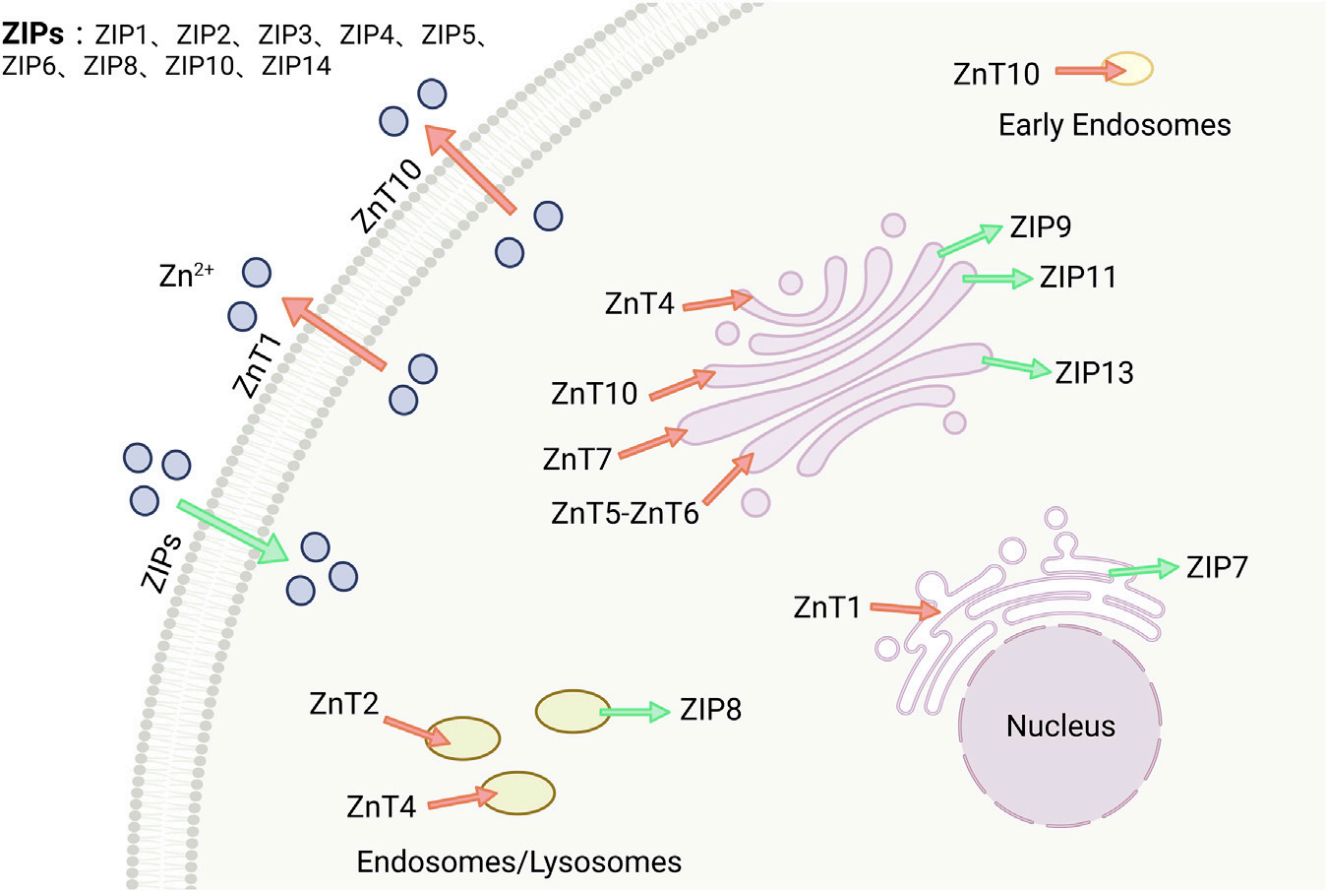
Zinc is maintained by two families, ZnTs and ZIPs. There are two families of zinc transport proteins that maintain the stability of zinc ions within the body. ZIPs (SLC39A) increase the concentration of zinc in the cytoplasm, while ZnTs (SLC30A) decrease the concentration of zinc in the cytoplasm. Image from biorender.

Zinc homeostasis is regulated by multiple proteins at various levels. Zinc ion transporters are the most significant among these regulators, which can be classified into two families: ZnTs (SLC30) and ZIPs (SLC39) (Kambe et al., 2021), as shown in Figure 3. The ZIPs family increases zinc ion concentration in the cytoplasm by promoting extracellular uptake or release from subcellular organelles. Conversely, the ZnTs family reduces zinc ion concentration in the cytoplasm. Imbalances or changes in the expression and localization of these zinc ion transporters can lead to zinc ion imbalances, resulting in various severe pathophysiological stimuli within cells (Tuncay et al., 2019). The expression of zinc ion transporters ZIP7 and ZnT7 at the protein and RNA levels is relatively low in mammalian heart tissues. ZIP7 is widely expressed in human and mice tissues, while ZnT7 is abundant in the liver and small intestine (Kirschke and Huang, 2003; Huang et al., 2005).

Kirschke et al. and Turan et al. analyzed confocal images of cardiomyocyte using immunofluorescence microscopy and Huygens software, they found that ZIP7 was primarily located in the sarcoplasmic(endoplasmic) reticulum [S(E)R], with a small portion in the Golgi apparatus, but not in the nucleus(Kirschke and Huang, 2003; Turan, 2019). Similarly, Tuncay et al. examined ZIP7 and ZnT7 colocalization in S(E)R preparations isolated from rat cardiomyocyte and observed the same localization pattern (Tuncay et al., 2017). They also discovered that hyperglycemia-induced intracellular redistribution of free zinc led to an increased cytoplasmic [Zn^2+^]_i_ and decreased [Zn^2+^]_i_ in S(E)R through activation of CK2α-associated ZIP7 phosphorylation (Tuncay et al., 2017).

### 4.2 Metallothionein

Metallothionein (MT) is a low-molecular-weight protein rich in mercaptans, which functions as a scavenger of free radicals (Wang et al., 2001), It contains a high concentration of cysteine, which effectively protects cells and tissues from oxidative damage (Kang, 1999). There are four subtypes of MT: MT-I and MT-II are present in all tissues and organs of mammals, MT-III is unique to brain tissues, and MT-IV is found only in certain tissues containing lamellar squamous epithelial cells (Meloni et al., 2006). Cardio-specific overexpression of MT and catalase significantly inhibited acute (Kang et al., 1997) and chronic (Sun et al., 2001) cardiotoxicity induced by Dox, proved by characteristic histopathological, ultrastructural changes, and dysfunction. Cardiomyocyte apoptosis induced by Dox was significantly reduced in mice with cardiac-specific overexpression of MT (Kang et al., 2000). What’s more, Zinc-MT strongly inhibited lipid peroxidation when present during incubation (Thomas et al., 1986).

### 4.3 Combining therapies for cardiac toxicity treatment

Researchers have made many attempts and innovations to reduce the cardiac side effects during chemotherapy. For example, Y. Zhang et al. found zinc-selenium tea can effectively alleviate the extent of myocardial fiber disarray, rupture, and inflammatory cell infiltration induced by nonylphenol (Zhang et al., 2023b); although green tea has a similar effect, not as pronounced as zinc-selenium tea (Zhang et al., 2023b). P.K. Badkoobeh found that the antioxidant nano-zinc oxide (nZnO) has a cellular protective effect for Dox-induced male gonadal toxicity (Badkoobeh et al., 2013). Zinc finger protein 260 (Zfp260), also known as phenylephrine-induced complex-1 (PEX1), is an effector of α-1-adrenergic signaling in cardiac hypertrophy. It has been found that overexpression of Zfp260 can upregulate anti-apoptotic genes and reduce doxorubicin-induced apoptosis in primary cardiomyocyte (Li et al., 2022b). Researchers also evaluated the cardioprotective effects of zinc taurine solid dispersion in SD rats. After giving Dox treatment, zinc taurine was found to alleviate the decrease in blood pressure and left ventricular pressure caused by Dox (Wang et al., 2015). It also reduced serum Zn^2+^ and albumin levels and inhibited cardiomyocyte apoptosis (Wang et al., 2015). In addition, R. Wu et al. found that Zn^2+^-Curcumin supplementation significantly attenuates Dox-induced zinc imbalance, improves Dox-induced cardiac dysfunction, reduces myocardial injury (Wu et al., 2019).

## 5 Maintenance of calcium homeostasis and promoting effect on DIC and potential therapeutic direction

Calcium is a vital trace element that plays various roles in the human body, both structurally and functionally. The concentration of intracellular and extracellular calcium is strictly regulated through processes such as intestinal absorption, renal reabsorption, and bone exchange, which are controlled by a group of interacting hormones, including parathyroid hormone, parathyroid hormone-related peptides, and key receptors (Matikainen et al., 2021), These mechanisms ensure that blood calcium concentrations and systemic calcium ion balance are maintained within a narrow range (Matikainen et al., 2021). Calcium exists in two forms in the body: as an inactive binding form and as the highly active bivalent cation Ca^2+^.

### 5.1 Calcium homeostasis

**FIGURE 4:**
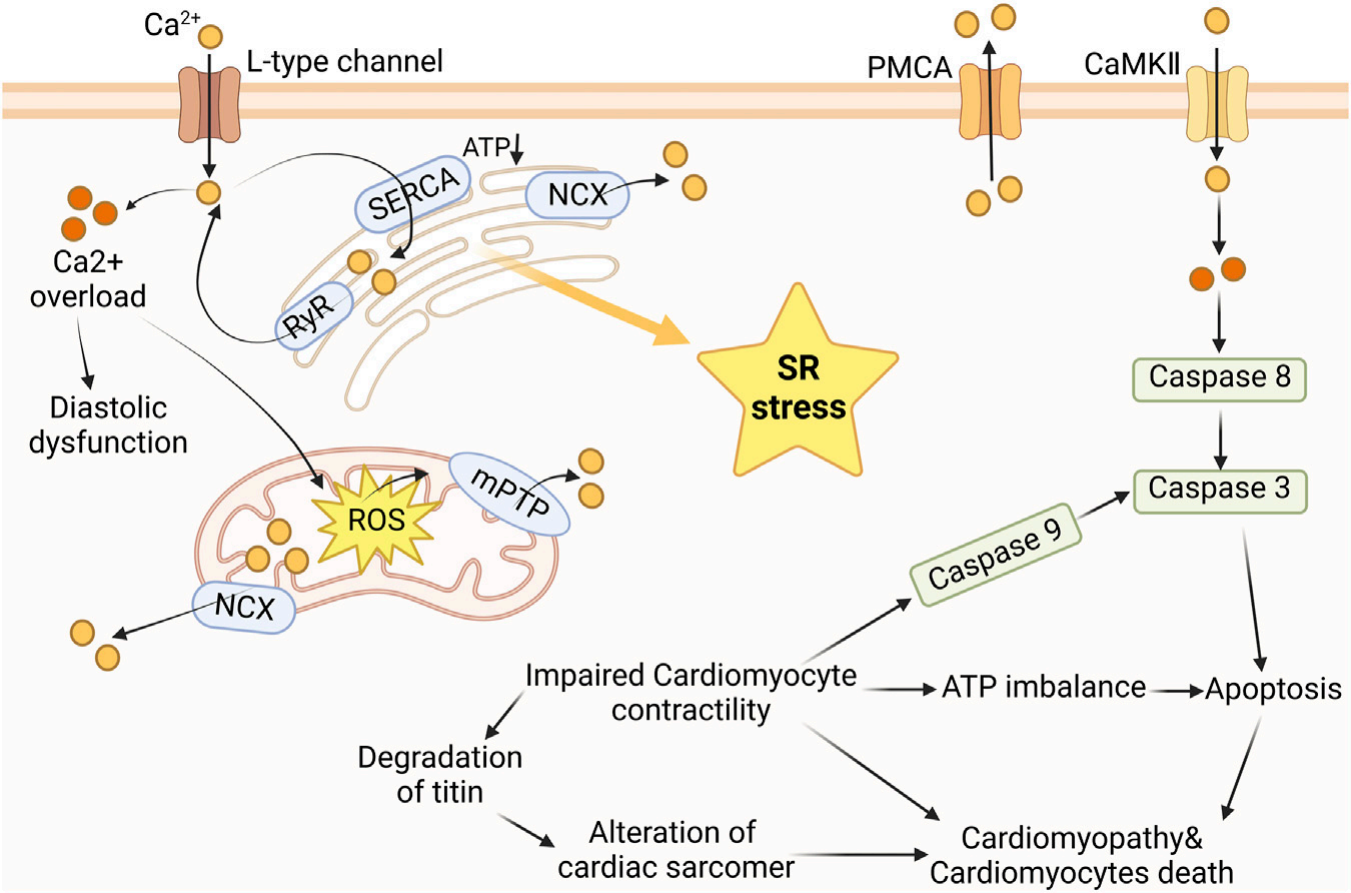
Calcium depends on the transport of different proteins between cytoplasm and organelles, and calcium overload promotes apoptosis. Ca^2+^ is pumped into the cytoplasm by L-Type channels and CaMKII. It then influences the calcium ion concentration in organelles such as mitochondria and endoplasmic reticulum through proteins like RyR, SERCA, and NCX. When there is calcium overload in cells, it results in endoplasmic reticulum stress and mitochondrial energy imbalance. This triggers the activation of apoptosis-related proteins, ultimately leading to myocardial disease. PMCA plasma membrane Ca^2+^-ATPases; CaMKII calcium/calmodulin-dependent protein kinase II; ATP adenosine triphosphate; SERCA sarcoendoplasmic reticulum calcium ATPase; RyR ryanodine receptor; NCX sodium (Na)-calcium exchanger; SR sarcoplasmic reticulum; mPTP mitochondrial permeability transition pore. Image from biorender.

In cardiomyocyte, calcium is predominantly found in the SR. According to Figure 4, under normal physiological conditions, the release of calcium ions from the SR generates ROS. However, under the influence of Dox, the release of Ca^2+^ increases, leading to apoptosis of cardiomyocyte. On the other hand, chelating agents of Ca^2+^ inhibit ROS production and cardiomyocyte apoptosis (Kalivendi et al., 2005). Disruption of Ca^2+^ balance triggers activation of the ER stress response. Maintaining Ca^2+^ homeostasis requires a highly integrated and complex system, which includes the plasma membrane Na^+^/Ca^2+^ exchanger (NCX), a system with low affinity but a strong capability to transport Ca^2+^. The plasma membrane Ca^2+^ pump (PMCA) and the S(E)R calcium ATPase (SERCA) have a high affinity for Ca^2+^ but poor capacity (Krebs et al., 2015). Similarly, in the case of DIC, ER stress can also induce cardiomyocyte apoptosis, further exacerbating the condition (Lakshmanan et al., 2013). Furthermore, during the metabolism of Dox, the toxic metabolite DOXOL is produced, which inhibits sodium/calcium exchange channels (Fu et al., 1990).

In a rabbit model with DIC, Arai et al. found that reduced expression levels of the SR mRNA gene responsible for calcium transport are the main cause of disrupt calcium homeostasis (Arai et al., 1998). Therefore, several studies have identified targets related to ER stress, aiming to expand the possibilities for preventing and treating DIC. For instance, it has been observed that Stim1 expression is downregulated in Dox-treated mice cardiomyocyte. Conversely, cardio-specific deficiency of Stim1 worsens Dox-induced cardiac dysfunction and cardiomyocyte apoptosis, while cardio-specific overexpression of Stim1 alleviates these phenomenon (Zhu et al., 2021); Additionally, downregulation of Stim1 enhances Dox-induced ER stress in cardiomyocyte, whereas overexpression of Stim1 inhibits the activation of molecular markers of ER stress (Zhu et al., 2021).

### 5.2 Calcium channel blockers

Dox also causes calcium overload in mitochondria. On one hand, Dox treatment can disrupt mitochondrial energy synthesis, and induce mutations and defects in mitochondrial DNA (Sheibani et al., 2022); On the other hand, elevated intracellular calcium concentration can also lead to mitochondrial dysfunction and apoptosis (Mitry and Edwards, 2016). To maintain the flow of Ca^2+^ across the mitochondrial membrane, Ca^2+^ enters the mitochondria through a specific non-adenosine triphosphate-dependent single transporter and then exits through Na^+^ carriers or Ca^2+^/H^+^ reverse transporters in the heart and liver, respectively (Mughal et al., 2018). Interestingly, calcium directly affects enzymes that stimulate the tricarboxylic acid cycle and the electron transport chain, resulting in increased oxidative phosphorylation (Ivannikov and Macleod, 2013). Mitochondria, in turn, regulate cellular calcium signaling by sequestering and buffering cytoplasmic calcium.

Calcium channel blockers can be used to prevent cellular death to alleviate calcium overload. Nicorandil, an indirect calcium channel blocker, can inhibit oxidative stress-induced apoptosis by opening mitochondrial KATP channels or activating NO/cGMP-dependent pathways (Nishikawa et al., 2006). Studies have shown that Nicorandil can preserve phosphocreatine and adenine nucleotide levels by restoring mitochondria’s oxidative phosphorylation ability and creatine kinase activity (Ahmed and El-Maraghy, 2013). Diltiazem (DIL), another calcium channel blocker, has been reported to downregulate the expression of the mitochondrial transporter ABCB1 gene in the human breast cancer cell line MCF-7 when used in combination with Dox. DIL treatment can reverse the resistance of breast cancer cells to Dox and has a protective effect against DIC (Al-Malky et al., 2019).

### 5.3 Calcium sensitizer

There are three overall types of calcium sensitizer agents (Pollesello et al., 2016). Type I calcium sensitizers increase the affinity of Ca^2+^ to troponin C either through direct action or conformational changes, enhancing myocardial contractility. Their mechanism of action is central, such as Pimobendan, CGP-48506, and Oxyphenamone. Type II calcium sensitizers act directly on cardiac myofilaments, promoting the regulation of actin and myosin, which increases the sensitivity of myocardial fibers to Ca^2+^ but does not affect the affinity of troponin C to Ca^2+^. Their mechanism of action is downstream, such as Levosimendan (LEVO). Type III calcium sensitizer directly acts on the troponin C and is considered a downstream mechanism. Moreover, it exhibits myocontractile effects by activating the actomyosin ATPase, even in the absence of Ca^2+^, such as EMD57033. While LEVO is one of the well-studied calcium sensitizer and vasodilator. Studies have shown that LEVO can activate the cAMP-PKA-PLN axis, reducing calcium overload in cardiomyocyte, and alleviating DIC (Efentakis et al., 2020). Additionally, LEVO can inhibit the activation of PTEN, promote the expression of P-Akt, decrease cell apoptosis, and repair cardiac function damage caused by Dox (Li et al., 2020a).

## 6 Discussion

All of the metals mentioned above, although the amount needed by the human body is very small, can cause signs of disease when absent. Abnormal transport of metal ions has been observed in patients with cardiomyopathy caused by continuous use of Dox in cancer treatment and in rodents with cumulative dosing of Dox to model cardiomyopathy. Previous literature suggests that overload and deposition of endogenous metal ions are part of the mechanism of DIC. An effective metal chelator should have the ability to cross the BBB, be specific to a single metal ion, and not interfere with the normal physiological metabolism of metal ions (Wang et al., 2020b). At present, metal chelators that satisfy these three conditions have not yet been identified for clinical treatment of DIC. Ferroptosis currently has only one clinical treatment option, which is Dexrazoxane. Studies have shown that Ferrostatin 1 can inhibit ferroptosis more than Dexrazoxane in mice (Fang et al., 2019). Interestingly, researchers have pointed out that the *in vivo* therapeutic function of Ferrostatin 1 is weaker than *in vitro* due to its instability in plasma. To tackle this, a soluble Ferrostatin analog named UAMC-3203 has been developed. UAMC-3203 is a more stable and effective inhibitor of ferroptosis (Jin et al., 2022a).

Currently, we have discovered various chemical drugs, natural compounds or traditional Chinese medicine formulations that exhibit different mechanisms in combating DIC. Examples of chemical compounds include empagliflozin (Quagliariello et al., 2021), sulforaphane (Wang et al., 2022b), dexmedetomidine (Wang et al., 2022d). Natural products such as puerarin (Huang et al., 2022), isoquercitrin (Luo et al., 2023), icariside II (Gao et al., 2023) have also shown efficacy. Additionally, traditional Chinese medicine compound formulations like qing-xin-jie-yu granule (Zhang et al., 2023a), kaixin san (Cao et al., 2023), and qishenyiqi drops (Wu et al., 2023) have demonstrated the ability to inhibit ferroptosis and maintain intracellular iron balance. Furthermore, supplementation with Ginkgolide B (Gao et al., 2016), vitamins B and D (Awad et al., 2021), Epalastat analogue NARI-29 (Syamprasad et al., 2023), and d-Limonene complexed with cyclodextrin (Durço et al., 2023) have been found to alleviate DIC through calcium-related pathways. Taurine zinc solid dispersions (Wang et al., 2015), Zn(ii)-curcumin supplementation (Wu et al., 2019), magnolol and honokiol complex (Aktay et al., 2023) have been shown to improve the abnormal state of Zn2+ during DIC. Interestingly, cannabidiol has the ability to restore normal calcium and zinc levels simultaneously, thereby playing a protective role for the heart (Fouad et al., 2013).

In addition to reducing DIC, metal ion overload also has certain cardiotoxic effects. For example, a series of previous meta-analysis studies conducted by Wang’s team found that an increase in dietary heme iron intake by 1 mg per day led to a 7% increase in the risk of heart disease (Fang et al., 2015). Furthermore, hereditary hemochromatosis, an autosomal recessive genetic disease characterized by large deposits of Fe^3+^ in multiple organs. According to a study by the Mayo Clinic in the United States, approximately one-third of hemochromatosis patients die from heart disease (Olson et al., 1987). Moreover, women with hemochromatosis have a lower risk of cardiovascular events than men, possibly due to persistent iron deficiency during women’s periods (Gaenzer et al., 2002). Similarly, uncombined or free copper is toxic, as almost all copper in the human body must be combined with copper proteins. Acute copper poisoning can lead to abdominal pain, nausea and vomiting, liver necrosis, kidney damage, and more. In terms of the cardiovascular system, it can lead to atherosclerosis. Excessive zinc or calcium in adults can also increase the risk of coronary heart disease and arteriosclerosis. As summarized in Table 1, Dox promotes apoptosis and mortality rates, induces myocardial diseases. However, when administered with metal ion inhibitors through various mechanisms, it exhibits significant cardiac protective effects. Metal ions have characteristics of a “double-edged sword,” so it is important to avoid excessive adjustment of their concentration.

**TABLE 1:** Potential therapeutic interventions in Dox-induced cardiotoxicity *in vivo/vitro*.

Metal ion species	Study model	Doxorubicin dosage and route of administration	Therapeutic intervention	Outcomes of therapy	References
Iron	*In vivo*/Ripk3^−/−^ mice or wild-type mice	20 mg/kg, ip, single dose	Ferrostatin 1 (1 mg/kg, ip)	↓ Dox-induced cell mortality	Fang et al. (2019)
				↓ Anp, Bnp and Myh7 mRNA levels	
				↑ LVEF, LVFS	
	*In vivo*/C75BL/6 mice	10 mg/kg, ip, single dose	ZnPP (10 mg/kg, ip)	↓ Ptgs2	Fang et al. (2019)
				↓ MDA	
				↓ Anp, Bnp and Myh7 mRNA levels	
	*In vivo*/Sprague-Dawley rats	6 mg/kg, iv, single dose	Feed iron-rich chow	↑ weight loss	Panjrath et al. (2007)
				↑ annexin uptake	
				↑ Cardiomyocyte damage	
	*In vivo*/C57BL/6 mice and neonatal Sprague Dawley rats, and *in vitro*/H9c2 cardiac cells	1 μM, 24 h 2.5 mg/kg, ip, 2 times per week for 3 weeks	Epigallocatechin-3-gallate (20 mg/kg/d, ig, for six consecutive weeks)	↓ CK-MB, LDH	He et al. (2021)
				↑ LVEF, LVFS	
				↑ LC3	
				↓ P62	
				↑ AMPKα2	
	*In vivo*/Hfe^−/−^mice or wild-type Hfe mice, and *in vitro*/H9c2 rat cardiomyocyte	10 μM, 24 h 20 mg/kg, ip, single dose	ABCB8 overexpression	↑ ABCB8	Menon and Kim (2022)
				↓ Dox retention	
				↓ mitochondrial iron	
				↓ oxidative stress	
	*In vivo*/Sprague-Dawley rats	15 mg/kg, ip, single dose	Deferiprone (10 mg/kg for 10 days, po, once daily for 10 days)	↓ Heart rate	El-Ammar el et al. (2011)
				↑ ST segment	
				↓ CK-MD	
				↓ LDH, MDA	
				↓ GSH, SOD	
	*In vivo*/Sprague-Dawley rats	2 mg/kg/d, ip, for a week	D-α-tocopherol succinate (2 g/kg, po, for a week)	↓ Weight gain	Berthiaume et al. (2005)
				↓ Heart weight	
				↑ the content of tocopherols	
	*In vivo*/TRIM21^−/−^ or TRIM21^+/+^ C57BL/6J mice and *in vitro*/mice embryonic fibroblasts and H9c cardiomyocyte line	20 mg/kg, ip, single dose	Loss of TRIM21	↑ Survival time	Hou et al. (2021)
				↑ Cardiac function	
				↓ 4-HNE	
				↓ MDA	
				↓ Ferroptosis	
	*In vivo*/Wistar albino rats	15 mg/kg, ip, single dose	Hemin (2.5, 5, 10 mg/kg/d, ip, for a week)	↓ CK-MB, LDH, MDA	Refaie et al. (2021)
				↑ GSH	
				↑ Nrf-2, and HO-1 mRNA level	
				↓ NF-κB	
				↓ Cleaved caspase-3	
				↓ cardiac muscle fibers	
Copper	*In vivo*/Sprague-Dawley rats	1, 2, or 4 mg/kg, 1 time/week for 4 weeks	Copper deficiency	↑ the activity of Cu, Zn superoxide dismutase, and glutathione S-transferase	Fischer et al. (1992)
	*In vitro*/HL/60 and its H_2_O_2_-resistant HP100 cells	1, 2, 5 μM, 3 h	H_2_O_2_-resistant	↑ 8-oxodG	Mizutani et al. (2003)
				↑ DNA damage	
Zinc	*In vitro*/ATCC CRL1446 and H9c2 cardiomyocyte	1 μM for 24 h	PKC inhibitor	↑ intracellular-free Zn^2+^	Olgar et al. (2018)
				↑ PUMA protein levels	
				↑ ER	
	*In vivo*/cardiac MT-overexpressing transgenic mice or normal controls	20 mg/kg, ip, single dose	Metallothionein overexpression	Maintain the normal morphology of cardiomyocyte	Kang et al. (1997)
				↓ serum CPK activity	
				↓ inotropy (left atrium)	
	*In vivo*/cardiac-specific overexpression of MT transgenic mice or nontransgenic controls	4 mg/kg, ip, 10 times in 7 weeks	Metallothionein overexpression	↓ Cardiac hypertrophy	Sun et al. (2001)
				↓ Dox-induced myocardial injury	
				↓ The degree of cytoplasmic vacuolation retains the fine ultrastructure of mitochondria	
	*In vivo*/cardiac-specific MT-overexpressing transgenic positive and negative FVB mice and *in vitro*/neonatal mice primary cardiomyocyte	15 mg/kg, ip, single dose 0.1, 0.5, 1.0 mM, 6 h	Metallothionein overexpression	↑ TUNEL-positive myocardium	Kang et al. (2000)
				↑ Morphological changes	
	*In vivo*/Adult male Wistar rats	6 mg/kg, ip, 3 d	Co-administration of nano-zinc oxide (5 mg/kg/d, 3 d)	↓ plasma total antioxidant power	Badkoobeh et al. (2013)
				↓ Lipid peroxidation	
				↓ plasma testosterone	
				↓ LH, Sperm count	
				↑ DNA damage	
	*In vivo*/Mature adult male Wistar rats	6 mg/kg, ip, 3 times weekly for 8 weeks	Co-administration of nano-zinc oxide (3 mg/kg/d, 5 times weekly for 8 weeks, po)	↓ GSH	El-Maddawy and Abd El Naby (2019)
				↓ CAT	
				↑ MDA	
				↓ reproductive organs	
				↓ epididymal sperm count	
				↓ live sperm	
	*In vivo*/C57BL/6 mice and *in votro*/Primary rat cardiomyocyte	15 mg/kg, ip, single dose 300 nM	Phenylephrine-induced complex-1 overexpression	↑ Cytoplasmic vacuolization and myofibril loss	Li et al. (2022b)
				↓ The transcript and protein levels of GATA-4	
				↓ Cell viability	
				↑ Apoptosis	
Calcium	*In vivo*/cardiomyocyte-specific stim1 knockout or WT C57BL/6 mice and *in vitro*/AC16 cardiomyocyte	200 μL, 15 mg/kg, at a rate of 0.5 μL/h, over a period of 14 days	STIM1 overexpression	↓ AC16 cardiomyocyte apoptosis	Zhu et al. (2021)
				↑ Cardiac function	
				↓ ER stress	
	*In vitro*/adult rat cardiomyocyte	3 µM	Ryanodine (20 µM)	↓ Dox-mediated SR Ca^2+^ release	Kim et al. (2006)
			Dantrolene (2 µM)	↓ ROS generation	
			α-lipoic acid (100 µM)	↓ Caspase 3 activity	
	*In vivo*/Wistar rats	3 mg/kg, ip, 3 times weekly(every other day), for a cumulative dose of 18 mg/kg	Nicorandil (3 mg/kg, po, over a period of 2 weeks)	↓ Rats mortality	Ahmed and El-Maraghy (2013)
				↑ Heart rate	
				↓ Oxidative stress, apoptosis	
				↓ the mitochondrial overall injury score	
	*In vivo* */*Wistar rats	15 mg/kg, ip, single dose	Diltiazem (4 mg/kg, ip, single dose)	↓ ABCB1 mRNA level	Al-Malky et al. (2019)
	*In vitro*/human breast cancer cell line MCF-7	0.25 or 1 μg/mL, 48 h	Diltiazem (20 μg/mL, 48 h)	↑ FOXO3a, P53	
				↓ CK-MB, MDA	
				↑ TAC, GPx	
	Protocol 1: *In vivo*/Wistar rats	Protocol 1: 20 mg/kg, ip, single dose	Protocol 1: Levosimendan (12 or 24 μg/kg, ip, single dose)	↑ Myocardial contractility	Efentakis et al. (2020)
	Protocol 2: *In vivo*/PLN^−/−^ or normal SV129 mice	Protocol 2: 3 mg/kg, ip, 3 times weekly	Protocol 2: Levosimendan (24 μg/kg, ip, single dose)	↓ Myocardial fibrosis	
	Protocol 3: *In vivo*/C57BL/6 mice	Protocol 3: 3 mg/kg, ip, 3 times weekly	Protocol 3: Levosimendan (6 μg/kg, ip, for 4 times or 24 μg/kg, ip, single dose)	↓ Cardiac hypertrophy	
				↓ MDA	
				↓ ROS accumulation	
				↓ Dox-induced changes of iNOs and MnSOD	
				↑ Phosphorylation of eNOs and Akt	
				↑ cGMP	
	*In vivo*/C57L/6 mice and *in vitro*/H9c2 cells	5 mg/kg, ip, 1 time/week for 4 weeks 1 μmol/mL, 24 h	Levosimendan (1 mg/kg, po, once daily for 4 weeks) Levosimendan (10 μmol/mL, 2 h)	↑ LVEF, LVFS	Li et al. (2020a)
				↓ Cardiac dysfunction	
				↑ HW/TL	
				↓ Anp, Bnp mRNA levels	
				↓ BAX, c-caspase-3	
				↑ Bcl-2	
				↓ Apoptosis	

Despite its side and adverse effects, Dox remains a key drug in many cancer treatments. The mechanism of DIC is influenced by many factors. Depending on the mechanism, different strategies can be developed to prevent or reduce the adverse cardiotoxicity of Dox. Previous studies have shown that ADAR2 overexpression attenuates DIC by enhancing cardiac function and reducing apoptosis (Wu et al., 2022). Similarly, DIC can be alleviated by activating some classical signaling pathways such as cAMP/PKA/SIRT1 (Hu et al., 2020), or AKT/SIRT3/SOD2 (Liu et al., 2022). Recently, some potential DIC therapeutic targets have been newly discovered, such as PDE10A (Chen et al., 2023), Sestrin 2 (Wang et al., 2022a), FAM134B (Qu et al., 2022), TFEB (Chen et al., 2022), etc., which play a cardioprotective role in reducing apoptosis, improving cardiac function, reducing oxidative stress and endoplasmic reticulum stress, and promoting autophagy. In addition, some non-coding RNAs have been shown to play important roles in DIC, such as miR-128-3p (Zhao et al., 2019), miR-451 (Li et al., 2019), miR-152 (Zhang et al., 2021), miR-125b (Jin et al., 2022b), etc. Recently, there are also newly discovered drugs with evidence of reducing DIC, such as Di’ao Xinxuekang capsule (Li et al., 2022c), tanshinone I (Jiang et al., 2022), glycyrrhetinic acid (Cheng et al., 2022), and Berberine (Wang et al., 2023), etc.

This paper discusses various metal ion chelators, ionic carriers, metal complexes, and natural chemical products that can reduce ROS production by regulating endogenous metal ion homeostasis, reducing oxidative stress and mitochondrial dysfunction, and thereby alleviating the cardiac toxicity of Dox. In future studies, it is hoped that by further improving the structure and deficiencies of these drugs, they will not only be effective at the animal and cellular level, but can also be used clinically through drug clinical trials to prevent or treat DIC.

## References

[B1] Abe K. Ikeda M. Ide T. Tadokoro T. Miyamoto H. D. Furusawa S. (2022). Doxorubicin causes ferroptosis and cardiotoxicity by intercalating into mitochondrial DNA and disrupting Alas1-dependent heme synthesis. Sci. Signal 15, eabn8017. 10.1126/scisignal.abn8017 36318618

[B2] Ahmed L. A. El-Maraghy S. A. (2013). Nicorandil ameliorates mitochondrial dysfunction in doxorubicin-induced heart failure in rats: possible mechanism of cardioprotection. Biochem. Pharmacol. 86, 1301–1310. 10.1016/j.bcp.2013.07.005 23872193

[B3] Aktay I. Bitirim C. V. Olgar Y. Durak A. Tuncay E. Billur D. (2023). Cardioprotective role of a magnolol and honokiol complex in the prevention of doxorubicin-mediated cardiotoxicity in adult rats. Mol. Cell Biochem. 10.1007/s11010-023-04728-w 37074505

[B4] Al-Malky H. S. Osman A. M. Damanhouri Z. A. Alkreathy H. M. Al Aama J. Y. Ramadan W. S. (2019). Modulation of doxorubicin-induced expression of the multidrug resistance gene in breast cancer cells by diltiazem and protection against cardiotoxicity in experimental animals. Cancer Cell Int. 19, 191. 10.1186/s12935-019-0912-0 31367189 PMC6657176

[B5] Anderson M. E. (1998). Glutathione: an overview of biosynthesis and modulation. Chem. Biol. Interact. 111-112, 1–14. 10.1016/s0009-2797(97)00146-4 9679538

[B6] Angelova M. Asenova S. Nedkova V. Kolevakolarova R. (2011). Copper in the human organism. Trakia J. Sci.

[B7] Anjum R. Palanimuthu D. Kalinowski D. S. Lewis W. Park K. C. Kovacevic Z. (2019). Synthesis, characterization, and *in vitro* anticancer activity of copper and zinc bis(thiosemicarbazone) complexes. Inorg. Chem. 58, 13709–13723. 10.1021/acs.inorgchem.9b01281 31339305

[B8] Apresova M. A. Efremova I. E. Babayants A. A. Cheknev S. B. (2014). γ-Globulin fraction proteins and their metal complexes with copper cations in induction of IL-8 production. Bull. Exp. Biol. Med. 156, 823–825. 10.1007/s10517-014-2460-x 24824707

[B9] Arai M. Tomaru K. Takizawa T. Sekiguchi K. Yokoyama T. Suzuki T. (1998). Sarcoplasmic reticulum genes are selectively down-regulated in cardiomyopathy produced by doxorubicin in rabbits. J. Mol. Cell Cardiol. 30, 243–254. 10.1006/jmcc.1997.0588 9515001

[B10] Awad H. H. El-Derany M. O. Mantawy E. M. Michel H. E. El-Naa M. M. Salah El-Din R. A. (2021). Comparative study on beneficial effects of vitamins B and D in attenuating doxorubicin induced cardiotoxicity in rats: emphasis on calcium homeostasis. Biomed. Pharmacother. 140, 111679. 10.1016/j.biopha.2021.111679 34029952

[B11] Baba Y. Higa J. K. Shimada B. K. Horiuchi K. M. Suhara T. Kobayashi M. (2018). Protective effects of the mechanistic target of rapamycin against excess iron and ferroptosis in cardiomyocytes. Am. J. Physiol. Heart Circ. Physiol. 314, H659–H668. 10.1152/ajpheart.00452.2017 29127238 PMC5899260

[B12] Badkoobeh P. Parivar K. Badkoobeh P. K. Kalantar S. M. Hosseini S. D. Salabat A. (2013). Effect of nano-zinc oxide on doxorubicin-induced oxidative stress and sperm disorders in adult male Wistar rats. Iran. J. Reproductive Med. 11, 355–364. PMC3941413 24639766

[B13] Banci L. Bertini I. (2013). Metallomics and the cell: some definitions and general comments. Met. Ions Life Sci. 12, 1–13. 10.1007/978-94-007-5561-1_1 23595668

[B14] BayıR A. Kara H. KıYıCı A. OztüRK B. AkyüREK F. (2013). Levels of selenium, zinc, copper, and cardiac troponin I in serum of patients with acute coronary syndrome. Biol. Trace Elem. Res. 154, 352–356. 10.1007/s12011-013-9754-0 23904327

[B15] Berthiaume J. M. Oliveira P. J. Fariss M. W. Wallace K. B. (2005). Dietary vitamin E decreases doxorubicin-induced oxidative stress without preventing mitochondrial dysfunction. Cardiovasc Toxicol. 5, 257–267. 10.1385/ct:5:3:257 16244371

[B16] Bhuvanasundar R. John A. Sulochana K. N. Coral K. Deepa P. R. Umashankar V. (2014). A molecular model of human Lysyl Oxidase (LOX) with optimal copper orientation in the catalytic cavity for induced fit docking studies with potential modulators. Bioinformation 10, 406–412. 10.6026/97320630010406 25187679 PMC4135287

[B17] Brem S. Tsanaclis A. M. Zagzag D. (1990). Anticopper treatment inhibits pseudopodial protrusion and the invasive spread of 9L gliosarcoma cells in the rat brain. Neurosurgery 26, 391–396. 10.1097/00006123-199003000-00003 2320207

[B18] Brewer G. J. (2009). The risks of copper toxicity contributing to cognitive decline in the aging population and to Alzheimer's disease. J. Am. Coll. Nutr. 28, 238–242. 10.1080/07315724.2009.10719777 20150596

[B19] Cao Y. Li M. Gu L. Zhao X. Zhou A. Miao Y. (2023). Chinese traditional formula Kaixin San suppressed ferroptosis of hippocampal neurons and cardiomyocytes in mice with paradoxical sleep deprivation. J. Ethnopharmacol. 304, 116034. 10.1016/j.jep.2022.116034 36529245

[B20] Cater M. A. Haupt Y. (2011). Clioquinol induces cytoplasmic clearance of the X-linked inhibitor of apoptosis protein (XIAP): therapeutic indication for prostate cancer. Biochem. J. 436, 481–491. 10.1042/BJ20110123 21426304

[B21] Cater M. A. Pearson H. B. Wolyniec K. Klaver P. Bilandzic M. Paterson B. M. (2013). Increasing intracellular bioavailable copper selectively targets prostate cancer cells. ACS Chem. Biol. 8, 1621–1631. 10.1021/cb400198p 23656859

[B22] Chang H. C. Wu R. Shang M. Sato T. Chen C. Shapiro J. S. (2016). Reduction in mitochondrial iron alleviates cardiac damage during injury. EMBO Mol. Med. 8, 247–267. 10.15252/emmm.201505748 26896449 PMC4772952

[B23] Chauhan M. Banerjee K. Arjmand F. (2007). DNA binding studies of novel Copper(II) complexes containing L-tryptophan as chiral auxiliary: *in vitro* antitumor activity of Cu-Sn2 complex in human neuroblastoma cells. Inorg. Chem. 46, 3072–3082. 10.1021/ic061753a 17378549

[B24] Chen D. Cui Q. C. Yang H. Barrea R. A. Sarkar F. H. Sheng S. (2007). Clioquinol, a therapeutic agent for Alzheimer's disease, has proteasome-inhibitory, androgen receptor-suppressing, apoptosis-inducing, and antitumor activities in human prostate cancer cells and xenografts. Cancer Res. 67, 1636–1644. 10.1158/0008-5472.CAN-06-3546 17308104

[B25] Chen D. Yu W. Zhong C. Hong Q. Huang G. Que D. (2022). Elabela ameliorates doxorubicin-induced cardiotoxicity by promoting autophagic flux through TFEB pathway. Pharmacol. Res. 178, 106186. 10.1016/j.phrs.2022.106186 35306141

[B26] Cheng Y. Wu X. Nie X. Wu Y. Zhang C. Lee S. M. (2022). Natural compound glycyrrhetinic acid protects against doxorubicin-induced cardiotoxicity by activating the Nrf2/HO-1 signaling pathway. Phytomedicine 106, 154407. 10.1016/j.phymed.2022.154407 36070662

[B27] Chen S. Chen J. Du W. Mickelsen D. M. Shi H. Yu H. (2023). PDE10A inactivation prevents doxorubicin-induced cardiotoxicity and tumor growth. Circ. Res. 133, 138–157. 10.1161/CIRCRESAHA.122.322264 37232184 PMC10428174

[B28] Cox C. Teknos T. N. Barrios M. Brewer G. J. Dick R. D. Merajver S. D. (2001). The role of copper suppression as an antiangiogenic strategy in head and neck squamous cell carcinoma. Laryngoscope 111, 696–701. 10.1097/00005537-200104000-00024 11359142

[B29] Crowe A. Jackaman C. Beddoes K. M. Ricciardo B. Nelson D. J. (2013). Rapid copper acquisition by developing murine mesothelioma: decreasing bioavailable copper slows tumor growth, normalizes vessels and promotes T cell infiltration. PLoS One 8, e73684. 10.1371/journal.pone.0073684 24013775 PMC3754934

[B30] Dabravolski S. A. Sadykhov N. K. Kartuesov A. G. Borisov E. E. Sukhorukov V. N. Orekhov A. N. (2022). Interplay between Zn(2+) homeostasis and mitochondrial functions in cardiovascular diseases and heart ageing. Int. J. Mol. Sci. 23, 6890. 10.3390/ijms23136890 35805904 PMC9266371

[B31] Denoyer D. Masaldan S. La Fontaine S. Cater M. A. (2015). Targeting copper in cancer therapy: 'Copper that Cancer. Metallomics 7, 1459–1476. 10.1039/c5mt00149h 26313539

[B32] Dikalova A. E. Bikineyeva A. T. Budzyn K. Nazarewicz R. R. Mccann L. Lewis W. (2010). Therapeutic targeting of mitochondrial superoxide in hypertension. Circ. Res. 107, 106–116. 10.1161/CIRCRESAHA.109.214601 20448215 PMC2901409

[B33] Dinkova-Kostova A. T. Talalay P. (2008). Direct and indirect antioxidant properties of inducers of cytoprotective proteins. Mol. Nutr. Food Res. 52 (1), S128–S138. 10.1002/mnfr.200700195 18327872

[B34] Dolma S. Lessnick S. L. Hahn W. C. Stockwell B. R. (2003). Identification of genotype-selective antitumor agents using synthetic lethal chemical screening in engineered human tumor cells. Cancer Cell 3, 285–296. 10.1016/s1535-6108(03)00050-3 12676586

[B35] DurçO A. O. Souza D. S. Rhana P. Costa A. D. Marques L. P. Santos L. (2023). d-Limonene complexed with cyclodextrin attenuates cardiac arrhythmias in an experimental model of doxorubicin-induced cardiotoxicity: possible involvement of calcium/calmodulin-dependent protein kinase type II. Toxicol. Appl. Pharmacol. 474, 116609. 10.1016/j.taap.2023.116609 37392997

[B36] Efentakis P. Varela A. Chavdoula E. Sigala F. Sanoudou D. Tenta R. (2020). Levosimendan prevents doxorubicin-induced cardiotoxicity in time- and dose-dependent manner: implications for inotropy. Cardiovasc Res. 116, 576–591. 10.1093/cvr/cvz163 31228183

[B37] El-Ammar S. M. Said S. A. Suddek G. M. El-Damarawy S. L. (2011). Amelioration of doxorubicin-induced cardiotoxicity by deferiprone in rats. Can. J. Physiol. Pharmacol. 89, 269–276. 10.1139/y11-020 21526973

[B38] El-Maddawy Z. K. Abd El Naby W. S. H. (2019). Protective effects of zinc oxide nanoparticles against doxorubicin induced testicular toxicity and DNA damage in male rats. Toxicol. Res. (Camb) 8, 654–662. 10.1039/c9tx00052f 31588342 PMC6762007

[B39] Ewer M. S. Lippman S. M. (2005). Type II chemotherapy-related cardiac dysfunction: time to recognize a new entity. J. Clin. Oncol. 23, 2900–2902. 10.1200/JCO.2005.05.827 15860848

[B40] Fang X. An P. Wang H. Wang X. Shen X. Li X. (2015). Dietary intake of heme iron and risk of cardiovascular disease: a dose-response meta-analysis of prospective cohort studies. Nutr. Metab. Cardiovasc Dis. 25, 24–35. 10.1016/j.numecd.2014.09.002 25439662

[B41] Fang X. Wang H. Han D. Xie E. Yang X. Wei J. (2019). Ferroptosis as a target for protection against cardiomyopathy. Proc. Natl. Acad. Sci. U. S. A. 116, 2672–2680. 10.1073/pnas.1821022116 30692261 PMC6377499

[B42] Fischer J. G. Tackett R. L. Howerth E. W. Johnson M. A. (1992). Copper deficient rat heart can compensate for doxorubicin-induced oxidant stress. Biol. Trace Elem. Res. 37, 233–251. 10.1007/bf02783798 7688536

[B43] Fischer J. G. Tackett R. L. Howerth E. W. Johnson M. A. (1993). Copper deficient rat heart can compensate for doxorubicin-induced oxidant stress. Biol. Trace Elem. Res. 37, 233–251. 10.1007/BF02783798 7688536

[B44] Fisher G. L. Byers V. S. Shifrine M. Levin A. S. (1976). Copper and zinc levels in serum from human patients with sarcomas. Cancer 37, 356–363. 10.1002/1097-0142(197601)37:1<356::aid-cncr2820370146>3.0.co;2-w 764964

[B45] Forcina G. C. Dixon S. J. (2019). GPX4 at the crossroads of lipid homeostasis and ferroptosis. Proteomics 19, e1800311. 10.1002/pmic.201800311 30888116

[B46] Fouad A. A. Albuali W. H. Al-Mulhim A. S. Jresat I. (2013). Cardioprotective effect of cannabidiol in rats exposed to doxorubicin toxicity. Environ. Toxicol. Pharmacol. 36, 347–357. 10.1016/j.etap.2013.04.018 23721741

[B47] Friedmann Angeli J. P. Schneider M. Proneth B. Tyurina Y. Y. Tyurin V. A. Hammond V. J. (2014). Inactivation of the ferroptosis regulator Gpx4 triggers acute renal failure in mice. Nat. Cell Biol. 16, 1180–1191. 10.1038/ncb3064 25402683 PMC4894846

[B48] Fu L. X. Waagstein F. Hjalmarson A. (1990). A new insight into adriamycin-induced cardiotoxicity. Int. J. Cardiol. 29, 15–20. 10.1016/0167-5273(90)90267-9 2262210

[B49] Gaenzer H. Marschang P. Sturm W. Neumayr G. Vogel W. Patsch J. (2002). Association between increased iron stores and impaired endothelial function in patients with hereditary hemochromatosis. J. Am. Coll. Cardiol. 40, 2189–2194. 10.1016/s0735-1097(02)02611-6 12505233

[B50] Gao J. Chen T. Zhao D. Zheng J. Liu Z. (2016). Ginkgolide B exerts cardioprotective properties against doxorubicin-induced cardiotoxicity by regulating reactive oxygen species, Akt and calcium signaling pathways *in vitro* and *in vivo* . PLoS One 11, e0168219. 10.1371/journal.pone.0168219 27973574 PMC5156426

[B51] Gao J. Ma C. Xia D. Chen N. Zhang J. Xu F. (2023). Icariside II preconditioning evokes robust neuroprotection against ischaemic stroke, by targeting Nrf2 and the OXPHOS/NF-κB/ferroptosis pathway. Br. J. Pharmacol. 180, 308–329. 10.1111/bph.15961 36166825

[B52] Guha S. Roy S. (2021). Enhanced expression of SLC4A11 by tert-Butylhydroquinone is mediated by direct binding of Nrf2 to the promoter of SLC4A11. Free Radic. Biol. Med. 167, 299–306. 10.1016/j.freeradbiomed.2021.03.006 33744340

[B53] Gupte A. Mumper R. J. (2009). Elevated copper and oxidative stress in cancer cells as a target for cancer treatment. Cancer Treat. Rev. 35, 32–46. 10.1016/j.ctrv.2008.07.004 18774652

[B54] Gutteridge J. M. (1984). Lipid peroxidation and possible hydroxyl radical formation stimulated by the self-reduction of a doxorubicin-iron (III) complex. Biochem. Pharmacol. 33, 1725–1728. 10.1016/0006-2952(84)90340-x 6329216

[B55] Hamilton S. Terentyev D. (2019). Altered intracellular calcium homeostasis and arrhythmogenesis in the aged heart. Int. J. Mol. Sci. 20, 2386. 10.3390/ijms20102386 31091723 PMC6566636

[B56] He H. Wang L. Qiao Y. Yang B. Yin D. He M. (2021). Epigallocatechin-3-gallate pretreatment alleviates doxorubicin-induced ferroptosis and cardiotoxicity by upregulating AMPKα2 and activating adaptive autophagy. Redox Biol. 48, 102185. 10.1016/j.redox.2021.102185 34775319 PMC8600154

[B57] Hou K. Shen J. Yan J. Zhai C. Zhang J. Pan J. A. (2021). Loss of TRIM21 alleviates cardiotoxicity by suppressing ferroptosis induced by the chemotherapeutic agent doxorubicin. EBioMedicine 69, 103456. 10.1016/j.ebiom.2021.103456 34233258 PMC8261003

[B58] Hu J. Y. Zhang D. L. Liu X. L. Li X. S. Cheng X. Q. Chen J. (2017). Pathological concentration of zinc dramatically accelerates abnormal aggregation of full-length human Tau and thereby significantly increases Tau toxicity in neuronal cells. Biochim. Biophys. Acta Mol. Basis Dis. 1863, 414–427. 10.1016/j.bbadis.2016.11.022 27890528

[B59] Huang L. Kirschke C. P. Zhang Y. Yu Y. Y. (2005). The ZIP7 gene (Slc39a7) encodes a zinc transporter involved in zinc homeostasis of the Golgi apparatus. J. Biol. Chem. 280, 15456–15463. 10.1074/jbc.M412188200 15705588

[B60] Huang Y. Wu H. Hu Y. Zhou C. Wu J. Wu Y. (2022). Puerarin attenuates oxidative stress and ferroptosis via AMPK/PGC1α/Nrf2 pathway after subarachnoid hemorrhage in rats. Antioxidants (Basel) 11, 1259. 10.3390/antiox11071259 35883750 PMC9312059

[B61] Hu C. Zhang X. Song P. Yuan Y. P. Kong C. Y. Wu H. M. (2020). Meteorin-like protein attenuates doxorubicin-induced cardiotoxicity via activating cAMP/PKA/SIRT1 pathway. Redox Biol. 37, 101747. 10.1016/j.redox.2020.101747 33045622 PMC7558217

[B62] Ichikawa Y. Ghanefar M. Bayeva M. Wu R. Khechaduri A. Naga Prasad S. V. (2014). Cardiotoxicity of doxorubicin is mediated through mitochondrial iron accumulation. J. Clin. Invest. 124, 617–630. 10.1172/JCI72931 24382354 PMC3904631

[B63] Ishida S. Andreux P. Poitry-Yamate C. Auwerx J. Hanahan D. (2013). Bioavailable copper modulates oxidative phosphorylation and growth of tumors. Proc. Natl. Acad. Sci. U. S. A. 110, 19507–19512. 10.1073/pnas.1318431110 24218578 PMC3845132

[B64] Ivannikov M. V. Macleod G. T. (2013). Mitochondrial free Ca^2^⁺ levels and their effects on energy metabolism in Drosophila motor nerve terminals. Biophys. J. 104, 2353–2361. 10.1016/j.bpj.2013.03.064 23746507 PMC3672877

[B65] Jiang Q. Chen X. Tian X. Zhang J. Xue S. Jiang Y. (2022). Tanshinone I inhibits doxorubicin-induced cardiotoxicity by regulating Nrf2 signaling pathway. Phytomedicine 106, 154439. 10.1016/j.phymed.2022.154439 36108374

[B66] Jiang X. Stockwell B. R. Conrad M. (2021). Ferroptosis: mechanisms, biology and role in disease. Nat. Rev. Mol. Cell Biol. 22, 266–282. 10.1038/s41580-020-00324-8 33495651 PMC8142022

[B67] Jin T. He Q. Cheng C. Li H. Liang L. Zhang G. (2022a). UAMC-3203 or/and deferoxamine improve post-resuscitation myocardial dysfunction through suppressing ferroptosis in a rat model of cardiac arrest. Shock 57, 344–350. 10.1097/SHK.0000000000001869 34618729 PMC8868183

[B68] Jin X. Yu W. Ye P. (2022b). MiR-125b enhances doxorubicin-induced cardiotoxicity by suppressing the nucleus-cytoplasmic translocation of YAP via targeting STARD13. Environ. Toxicol. 37, 730–740. 10.1002/tox.23438 34921586

[B69] Johansson B. (1992). A review of the pharmacokinetics and pharmacodynamics of disulfiram and its metabolites. Acta Psychiatr. Scand. Suppl. 369, 15–26. 10.1111/j.1600-0447.1992.tb03310.x 1471547

[B70] Kalivendi S. V. Konorev E. A. Cunningham S. Vanamala S. K. Kaji E. H. Joseph J. (2005). Doxorubicin activates nuclear factor of activated T-lymphocytes and Fas ligand transcription: role of mitochondrial reactive oxygen species and calcium. Biochem. J. 389, 527–539. 10.1042/BJ20050285 15799720 PMC1175131

[B71] Kambe T. Taylor K. M. Fu D. (2021). Zinc transporters and their functional integration in mammalian cells. J. Biol. Chem. 296, 100320. 10.1016/j.jbc.2021.100320 33485965 PMC7949119

[B72] Kang Y. J. (1999). The antioxidant function of metallothionein in the heart. Proc. Soc. Exp. Biol. Med. 222, 263–273. 10.1046/j.1525-1373.1999.d01-143.x 10601885

[B73] Kang Y. J. Chen Y. Yu A. Voss-Mccowan M. Epstein P. N. (1997). Overexpression of metallothionein in the heart of transgenic mice suppresses doxorubicin cardiotoxicity. J. Clin. Invest. 100, 1501–1506. 10.1172/JCI119672 9294117 PMC508330

[B74] Kang Y. J. Zhou Z. X. Wang G. W. Buridi A. Klein J. B. (2000). Suppression by metallothionein of doxorubicin-induced cardiomyocyte apoptosis through inhibition of p38 mitogen-activated protein kinases. J. Biol. Chem. 275, 13690–13698. 10.1074/jbc.275.18.13690 10788488

[B75] Kim D. H. Kim W. D. Kim S. K. Moon D. H. Lee S. J. (2020). TGF-β1-mediated repression of SLC7A11 drives vulnerability to GPX4 inhibition in hepatocellular carcinoma cells. Cell Death Dis. 11, 406. 10.1038/s41419-020-2618-6 32471991 PMC7260246

[B76] Kim S. Y. Kim S. J. Kim B. J. Rah S. Y. Chung S. M. Im M. J. (2006). Doxorubicin-induced reactive oxygen species generation and intracellular Ca2+ increase are reciprocally modulated in rat cardiomyocytes. Exp. Mol. Med. 38, 535–545. 10.1038/emm.2006.63 17079870

[B77] Kim J. Kundu M. Viollet B. Guan K. L. (2011). AMPK and mTOR regulate autophagy through direct phosphorylation of Ulk1. Nat. Cell Biol. 13, 132–141. 10.1038/ncb2152 21258367 PMC3987946

[B78] King J. C. Shames D. M. Woodhouse L. R. (2000). Zinc homeostasis in humans. J. Nutr. 130, 1360S–6s. 10.1093/jn/130.5.1360S 10801944

[B79] Kirschke C. P. Huang L. (2003). ZnT7, a novel mammalian zinc transporter, accumulates zinc in the Golgi apparatus. J. Biol. Chem. 278, 4096–4102. 10.1074/jbc.M207644200 12446736

[B80] Kitakata H. Endo J. Ikura H. Moriyama H. Shirakawa K. Katsumata Y. (2022). Therapeutic targets for DOX-induced cardiomyopathy: role of apoptosis vs. Ferroptosis. Int. J. Mol. Sci. 23, 1414. 10.3390/ijms23031414 35163335 PMC8835899

[B81] Komatsu Y. Sadakata I. Ogra Y. Suzuki K. T. (2000). Excretion of copper complexed with thiomolybdate into the bile and blood in LEC rats. Chem. Biol. Interact. 124, 217–231. 10.1016/s0009-2797(99)00159-3 10728780

[B82] Krebs J. Agellon L. B. Michalak M. (2015). Ca(2+) homeostasis and endoplasmic reticulum (ER) stress: an integrated view of calcium signaling. Biochem. Biophys. Res. Commun. 460, 114–121. 10.1016/j.bbrc.2015.02.004 25998740

[B83] Lakshmanan A. P. Harima M. Suzuki K. Soetikno V. Nagata M. Nakamura T. (2013). The hyperglycemia stimulated myocardial endoplasmic reticulum (ER) stress contributes to diabetic cardiomyopathy in the transgenic non-obese type 2 diabetic rats: a differential role of unfolded protein response (UPR) signaling proteins. Int. J. Biochem. Cell Biol. 45, 438–447. 10.1016/j.biocel.2012.09.017 23032698

[B84] Lewerenz J. Hewett S. J. Huang Y. Lambros M. Gout P. W. Kalivas P. W. (2013). The cystine/glutamate antiporter system x(c)(-) in health and disease: from molecular mechanisms to novel therapeutic opportunities. Antioxid. Redox Signal 18, 522–555. 10.1089/ars.2011.4391 22667998 PMC3545354

[B85] Li D. L. Hill J. A. (2014). Cardiomyocyte autophagy and cancer chemotherapy. J. Mol. Cell Cardiol. 71, 54–61. 10.1016/j.yjmcc.2013.11.007 24239608 PMC4011970

[B86] Li L. L. Wei L. Zhang N. Wei W. Y. Hu C. Deng W. (2020a). Levosimendan protects against doxorubicin-induced cardiotoxicity by regulating the PTEN/Akt pathway. Biomed. Res. Int. 2020, 8593617. 10.1155/2020/8593617 32596387 PMC7298255

[B87] Li D. Song C. Zhang J. Zhao X. (2022a). ROS and iron homeostasis dependent ferroptosis play a vital role in 5-Fluorouracil induced cardiotoxicity *in vitro* and *in vivo* . Toxicology 468, 153113. 10.1016/j.tox.2022.153113 35101590

[B88] Li J. Wan W. Chen T. Tong S. Jiang X. Liu W. (2019). miR-451 silencing inhibited doxorubicin exposure-induced cardiotoxicity in mice. Biomed. Res. Int. 2019, 1528278. 10.1155/2019/1528278 31355248 PMC6637715

[B89] Li N. Wang W. Zhou H. Wu Q. Duan M. Liu C. (2020b). Ferritinophagy-mediated ferroptosis is involved in sepsis-induced cardiac injury. Free Radic. Biol. Med. 160, 303–318. 10.1016/j.freeradbiomed.2020.08.009 32846217

[B90] Lin H. Li N. He H. Ying Y. Sunkara S. Luo L. (2015). AMPK inhibits the stimulatory effects of TGF-β on smad2/3 activity, cell migration, and epithelial-to-mesenchymal transition. Mol. Pharmacol. 88, 1062–1071. 10.1124/mol.115.099549 26424816 PMC4658597

[B91] Liu X. Li D. Pi W. Wang B. Xu S. Yu L. (2022). LCZ696 protects against doxorubicin-induced cardiotoxicity by inhibiting ferroptosis via AKT/SIRT3/SOD2 signaling pathway activation. Int. Immunopharmacol. 113, 109379. 10.1016/j.intimp.2022.109379 36330913

[B92] Li W. Xie H. Hu H. Huang J. Chen S. (2022b). PEX1 is a mediator of α1-adrenergic signaling attenuating doxorubicin-induced cardiotoxicity. J. Biochem. Mol. Toxicol. 36, e23196. 10.1002/jbt.23196 35979984

[B93] Li X. Liang J. Qin A. Wang T. Liu S. Li W. (2022c). Protective effect of Di'ao Xinxuekang capsule against doxorubicin-induced chronic cardiotoxicity. J. Ethnopharmacol. 287, 114943. 10.1016/j.jep.2021.114943 34954266

[B94] Li Y. Chen F. Chen J. Chan S. He Y. Liu W. (2020c). Disulfiram/copper induces antitumor activity against both nasopharyngeal cancer cells and cancer-associated fibroblasts through ROS/MAPK and ferroptosis pathways. Cancers (Basel) 12, 138. 10.3390/cancers12010138 31935835 PMC7017005

[B95] Luo X. Gong Y. Jiang Q. Wang Q. Li S. Liu L. (2023). Isoquercitrin promotes ferroptosis and oxidative stress in nasopharyngeal carcinoma via the AMPK/NF-κB pathway. J. Biochem. Mol. Toxicol., e23542. 10.1002/jbt.23542 37712196

[B96] Mammoto T. Jiang A. Jiang E. Panigrahy D. Kieran M. W. Mammoto A. (2013). Role of collagen matrix in tumor angiogenesis and glioblastoma multiforme progression. Am. J. Pathol. 183, 1293–1305. 10.1016/j.ajpath.2013.06.026 23928381 PMC3791684

[B97] Marzano C. Pellei M. Tisato F. Santini C. (2009). Copper complexes as anticancer agents. Anticancer Agents Med. Chem. 9, 185–211. 10.2174/187152009787313837 19199864

[B98] Matikainen N. Pekkarinen T. Ryhanen E. M. Schalin-Jantti C. (2021). Physiology of calcium homeostasis: an overview. Endocrinol. Metab. Clin. North Am. 50, 575–590. 10.1016/j.ecl.2021.07.005 34774235

[B99] Meloni G. Zovo K. Kazantseva J. Palumaa P. VasáK M. (2006). Organization and assembly of metal-thiolate clusters in epithelium-specific metallothionein-4. J. Biol. Chem. 281, 14588–14595. 10.1074/jbc.M601724200 16556599

[B100] Meng H. Wang Y. Zhou F. Ruan J. Duan M. Wang X. (2021). Reduced serum zinc ion concentration is associated with coronary heart disease. Biol. Trace Elem. Res. 199, 4109–4118. 10.1007/s12011-020-02551-8 33387273

[B101] Menon A. V. Kim J. (2022). Iron promotes cardiac doxorubicin retention and toxicity through downregulation of the mitochondrial exporter ABCB8. Front. Pharmacol. 13, 817951. 10.3389/fphar.2022.817951 35359834 PMC8963208

[B102] Mitry M. A. Edwards J. G. (2016). Doxorubicin induced heart failure: phenotype and molecular mechanisms. Int. J. Cardiol. Heart Vasc. 10, 17–24. 10.1016/j.ijcha.2015.11.004 27213178 PMC4871279

[B103] Mizutani H. Oikawa S. Hiraku Y. Murata M. Kojima M. Kawanishi S. (2003). Distinct mechanisms of site-specific oxidative DNA damage by doxorubicin in the presence of copper(II) and NADPH-cytochrome P450 reductase. Cancer Sci. 94, 686–691. 10.1111/j.1349-7006.2003.tb01503.x 12901793 PMC11160291

[B104] Mughal W. Martens M. Field J. Chapman D. Huang J. Rattan S. (2018). Myocardin regulates mitochondrial calcium homeostasis and prevents permeability transition. Cell Death Differ. 25, 1732–1748. 10.1038/s41418-018-0073-z 29511336 PMC6180099

[B105] Mukhopadhyay P. Rajesh M. BáTKAI S. Kashiwaya Y. Haskó G. Liaudet L. (2009). Role of superoxide, nitric oxide, and peroxynitrite in doxorubicin-induced cell death *in vivo* and *in vitro* . Am. J. Physiol. Heart Circ. Physiol. 296, H1466–H1483. 10.1152/ajpheart.00795.2008 19286953 PMC2685360

[B106] Nishikawa S. Tatsumi T. Shiraishi J. Matsunaga S. Takeda M. Mano A. (2006). Nicorandil regulates Bcl-2 family proteins and protects cardiac myocytes against hypoxia-induced apoptosis. J. Mol. Cell Cardiol. 40, 510–519. 10.1016/j.yjmcc.2006.01.020 16527305

[B107] Olgar Y. Durak A. Tuncay E. Bitirim C. V. Ozcinar E. Inan M. B. (2018). Increased free Zn(2+) correlates induction of sarco(endo)plasmic reticulum stress via altered expression levels of Zn(2+) -transporters in heart failure. J. Cell Mol. Med. 22, 1944–1956. 10.1111/jcmm.13480 29333637 PMC5824399

[B108] Olson L. J. Edwards W. D. Mccall J. T. Ilstrup D. M. Gersh B. J. (1987). Cardiac iron deposition in idiopathic hemochromatosis: histologic and analytic assessment of 14 hearts from autopsy. J. Am. Coll. Cardiol. 10, 1239–1243. 10.1016/s0735-1097(87)80124-9 3680791

[B109] Panjrath G. S. Patel V. Valdiviezo C. I. Narula N. Narula J. Jain D. (2007). Potentiation of Doxorubicin cardiotoxicity by iron loading in a rodent model. J. Am. Coll. Cardiol. 49, 2457–2464. 10.1016/j.jacc.2007.02.060 17599610

[B110] Pan Q. Bao L. W. Merajver S. D. (2003). Tetrathiomolybdate inhibits angiogenesis and metastasis through suppression of the NFkappaB signaling cascade. Mol. Cancer Res. 1, 701–706. 12939395

[B111] Pivetta T. Lallai V. Valletta E. Trudu F. Isaia F. Perra D. (2015). Mixed copper-platinum complex formation could explain synergistic antiproliferative effect exhibited by binary mixtures of cisplatin and copper-1,10-phenanthroline compounds: an ESI-MS study. J. Inorg. Biochem. 151, 107–114. 10.1016/j.jinorgbio.2015.05.004 26021964

[B112] Pollesello P. Papp Z. Papp J. G. (2016). Calcium sensitizers: what have we learned over the last 25 years? Int. J. Cardiol. 203, 543–548. 10.1016/j.ijcard.2015.10.240 26580334

[B113] Purchase R. (2013). The link between copper and Wilson's disease. Sci. Prog. 96, 213–223. 10.3184/003685013X13712193905878 24244969 PMC10365353

[B114] Quagliariello V. De Laurentiis M. Rea D. Barbieri A. Monti M. G. Carbone A. (2021). The SGLT-2 inhibitor empagliflozin improves myocardial strain, reduces cardiac fibrosis and pro-inflammatory cytokines in non-diabetic mice treated with doxorubicin. Cardiovasc Diabetol. 20, 150. 10.1186/s12933-021-01346-y 34301253 PMC8305868

[B115] Qu Y. Gao R. Wei X. Sun X. Yang K. Shi H. (2022). Gasdermin D mediates endoplasmic reticulum stress via FAM134B to regulate cardiomyocyte autophagy and apoptosis in doxorubicin-induced cardiotoxicity. Cell Death Dis. 13, 901. 10.1038/s41419-022-05333-3 36289195 PMC9606128

[B116] Refaie M. M. M. Shehata S. Ibrahim R. A. Bayoumi A. M. A. Abdel-Gaber S. A. (2021). Dose-dependent cardioprotective effect of hemin in doxorubicin-induced cardiotoxicity via nrf-2/HO-1 and TLR-5/NF-κB/TNF-α signaling pathways. Cardiovasc Toxicol. 21, 1033–1044. 10.1007/s12012-021-09694-7 34510376

[B117] Ren J. Xu X. Wang Q. Ren S. Y. Dong M. Zhang Y. (2016). Permissive role of AMPK and autophagy in adiponectin deficiency-accentuated myocardial injury and inflammation in endotoxemia. J. Mol. Cell Cardiol. 93, 18–31. 10.1016/j.yjmcc.2016.02.002 26906634

[B118] Renu K. Abilash V. G. Tirupathi Pichiah P. B. Arunachalam S. (2018). Molecular mechanism of doxorubicin-induced cardiomyopathy - an update. Eur. J. Pharmacol. 818, 241–253. 10.1016/j.ejphar.2017.10.043 29074412

[B119] Schimmer A. D. Jitkova Y. Gronda M. Wang Z. Brandwein J. Chen C. (2012). A phase I study of the metal ionophore clioquinol in patients with advanced hematologic malignancies. Clin. Lymphoma Myeloma Leuk. 12, 330–336. 10.1016/j.clml.2012.05.005 22683301

[B120] Sheibani M. Azizi Y. Shayan M. Nezamoleslami S. Eslami F. Farjoo M. H. (2022). Doxorubicin-induced cardiotoxicity: an overview on pre-clinical therapeutic approaches. Cardiovasc Toxicol. 22, 292–310. 10.1007/s12012-022-09721-1 35061218

[B121] Sun L. Wang H. Yu S. Zhang L. Jiang J. Zhou Q. (2022). Herceptin induces ferroptosis and mitochondrial dysfunction in H9c2 cells. Int. J. Mol. Med. 12. 10.3892/ijmm.2021.5072 PMC8711589 34935058

[B122] Sun X. Zhou Z. Kang Y. J. (2001). Attenuation of doxorubicin chronic toxicity in metallothionein-overexpressing transgenic mouse heart. Cancer Res. 61, 3382–3387. 11309296

[B123] Swain S. M. Whaley F. S. Ewer M. S. (2003). Congestive heart failure in patients treated with doxorubicin: a retrospective analysis of three trials. Cancer 97, 2869–2879. 10.1002/cncr.11407 12767102

[B124] Syamprasad N. P. Jain S. Rajdev B. Panda S. R. Gangasani J. K. Challa V. S. (2023). AKR1B1 inhibition using NARI-29-an Epalrestat analogue-alleviates Doxorubicin-induced cardiotoxicity via modulating Calcium/CaMKII/MuRF-1 axis. Chem. Biol. Interact. 381, 110566. 10.1016/j.cbi.2023.110566 37257577

[B125] Tadokoro T. Ikeda M. Ide T. Deguchi H. Ikeda S. Okabe K. (2020). Mitochondria-dependent ferroptosis plays a pivotal role in doxorubicin cardiotoxicity. JCI Insight 5, e132747. 10.1172/jci.insight.132747 32376803 PMC7253028

[B126] Tainer J. A. Getzoff E. D. Richardson J. S. Richardson D. C. (1983). Structure and mechanism of copper, zinc superoxide dismutase. Nature 306, 284–287. 10.1038/306284a0 6316150

[B127] Thomas J. P. Bachowski G. J. Girotti A. W. (1986). Inhibition of cell membrane lipid peroxidation by cadmium- and zinc-metallothioneins. Biochim. Biophys. Acta 884, 448–461. 10.1016/0304-4165(86)90195-9 3778934

[B128] Townsend D. M. Tew K. D. Tapiero H. (2003). The importance of glutathione in human disease. Biomed. Pharmacother. 57, 145–155. 10.1016/s0753-3322(03)00043-x 12818476 PMC6522248

[B129] Tuncay E. Bitirim C. V. Olgar Y. Durak A. Rutter G. A. Turan B. (2019). Zn(2+)-transporters ZIP7 and ZnT7 play important role in progression of cardiac dysfunction via affecting sarco(endo)plasmic reticulum-mitochondria coupling in hyperglycemic cardiomyocytes. Mitochondrion 44, 41–52. 10.1016/j.mito.2017.12.011 29307859

[B130] Tuncay E. Bitirim V. C. Durak A. Carrat G. R. J. Taylor K. M. Rutter G. A. (2017). Hyperglycemia-induced changes in ZIP7 and ZnT7 expression cause Zn(2+) release from the sarco(endo)plasmic reticulum and mediate ER stress in the heart. Diabetes 66, 1346–1358. 10.2337/db16-1099 28232492

[B131] Turan B. (2019). A brief overview from the physiological and detrimental roles of zinc homeostasis via zinc transporters in the heart. Biol. Trace Elem. Res. 188, 160–176. 10.1007/s12011-018-1464-1 30091070

[B132] Uzel C. Conrad M. E. (1998). Absorption of heme iron. Semin. Hematol. 35, 27–34. 9460807

[B133] Wang A. J. Tang Y. Zhang J. Wang B. J. Xiao M. Lu G. (2022a). Cardiac SIRT1 ameliorates doxorubicin-induced cardiotoxicity by targeting sestrin 2. Redox Biol. 52, 102310. 10.1016/j.redox.2022.102310 35452917 PMC9043985

[B134] Wang G. W. Klein J. B. Kang Y. J. (2001). Metallothionein inhibits doxorubicin-induced mitochondrial cytochrome c release and caspase-3 activation in cardiomyocytes. J. Pharmacol. Exp. Ther. 298, 461–468. 11454906

[B135] Wang L. Liu Y. Du T. Yang H. Lei L. Guo M. (2020a). ATF3 promotes erastin-induced ferroptosis by suppressing system Xc^-^ . Cell Death Differ. 27, 662–675. 10.1038/s41418-019-0380-z 31273299 PMC7206049

[B136] Wang L. Yin Y. L. Liu X. Z. Shen P. Zheng Y. G. Lan X. R. (2020b). Current understanding of metal ions in the pathogenesis of Alzheimer's disease. Transl. Neurodegener. 9, 10. 10.1186/s40035-020-00189-z 32266063 PMC7119290

[B137] Wang X. Chen X. Zhou W. Men H. Bao T. Sun Y. (2022b). Ferroptosis is essential for diabetic cardiomyopathy and is prevented by sulforaphane via AMPK/NRF2 pathways. Acta Pharm. Sin. B 12, 708–722. 10.1016/j.apsb.2021.10.005 35256941 PMC8897044

[B138] Wang Y. Liao J. Luo Y. Li M. Su X. Yu B. (2023). Berberine alleviates doxorubicin-induced myocardial injury and fibrosis by eliminating oxidative stress and mitochondrial damage via promoting nrf-2 pathway activation. Int. J. Mol. Sci. 24, 3257. 10.3390/ijms24043257 36834687 PMC9966753

[B139] Wang Y. Mei X. Yuan J. Lu W. Li B. Xu D. (2015). Taurine zinc solid dispersions attenuate doxorubicin-induced hepatotoxicity and cardiotoxicity in rats. Toxicol. Appl. Pharmacol. 289, 1–11. 10.1016/j.taap.2015.08.017 26335259

[B140] Wang Y. Yan S. Liu X. Deng F. Wang P. Yang L. (2022c). PRMT4 promotes ferroptosis to aggravate doxorubicin-induced cardiomyopathy via inhibition of the Nrf2/GPX4 pathway. Cell Death Differ. 29, 1982–1995. 10.1038/s41418-022-00990-5 35383293 PMC9525272

[B141] Wang Z. Yao M. Jiang L. Wang L. Yang Y. Wang Q. (2022d). Dexmedetomidine attenuates myocardial ischemia/reperfusion-induced ferroptosis via AMPK/GSK-3β/Nrf2 axis. Biomed. Pharmacother. 154, 113572. 10.1016/j.biopha.2022.113572 35988428

[B142] Weiss G. Loyevsky M. Gordeuk V. R. (1999). Dexrazoxane (ICRF-187). Gen. Pharmacol. 32, 155–158. 10.1016/s0306-3623(98)00100-1 9888268

[B143] Wu L. Fan Z. Gu L. Liu J. Cui Z. Yu B. (2023). QiShenYiQi dripping pill alleviates myocardial ischemia-induced ferroptosis via improving mitochondrial dynamical homeostasis and biogenesis. J. Ethnopharmacol. 308, 116282. 10.1016/j.jep.2023.116282 36806343

[B144] Wu R. Mei X. Wang J. Sun W. Xue T. Lin C. (2019). Zn(ii)-Curcumin supplementation alleviates gut dysbiosis and zinc dyshomeostasis during doxorubicin-induced cardiotoxicity in rats. Food Funct. 10, 5587–5604. 10.1039/c9fo01034c 31432062

[B145] Wu X. Wang L. Wang K. Li J. Chen R. Wu X. (2022). ADAR2 increases in exercised heart and protects against myocardial infarction and doxorubicin-induced cardiotoxicity. Mol. Ther. 30, 400–414. 10.1016/j.ymthe.2021.07.004 34274534 PMC8753375

[B146] Ye Y. Chen A. Li L. Liang Q. Wang S. Dong Q. (2022). Repression of the antiporter SLC7A11/glutathione/glutathione peroxidase 4 axis drives ferroptosis of vascular smooth muscle cells to facilitate vascular calcification. Kidney Int. 102, 1259–1275. 10.1016/j.kint.2022.07.034 36063875

[B147] Yoshii J. Yoshiji H. Kuriyama S. Ikenaka Y. Noguchi R. Okuda H. (2001). The copper-chelating agent, trientine, suppresses tumor development and angiogenesis in the murine hepatocellular carcinoma cells. Int. J. Cancer 94, 768–773. 10.1002/ijc.1537 11745476

[B148] Zaidi Y. Arjmand F. Zaidi N. Usmani J. A. Zubair H. Akhtar K. (2014). A comprehensive biological insight of trinuclear copper(II)-tin(IV) chemotherapeutic anticancer drug entity: *in vitro* cytotoxicity and *in vivo* systemic toxicity studies. Metallomics 6, 1469–1479. 10.1039/c4mt00035h 24817323

[B149] Zhang W. B. Lai X. Guo X. F. (2021). Activation of Nrf2 by miR-152 inhibits doxorubicin-induced cardiotoxicity via attenuation of oxidative stress, inflammation, and apoptosis. Oxid. Med. Cell Longev. 2021, 8860883. 10.1155/2021/8860883 33574984 PMC7857911

[B150] Zhang J. Wang X. Guan B. Wang X. An X. Wang T. (2023a). Qing-Xin-Jie-Yu Granule inhibits ferroptosis and stabilizes atherosclerotic plaques by regulating the GPX4/xCT signaling pathway. J. Ethnopharmacol. 301, 115852. 10.1016/j.jep.2022.115852 36272494

[B151] Zhang Y. Xu J. Liu C. Long X. Zheng M. He J. (2023b). Curative effect of zinc-selenium tea on rat's cardiotoxicity induced by long-term exposure to nonylphenol. Environ. Toxicol. 38, 101–114. 10.1002/tox.23667 36239032

[B152] Zhao L. Zhou X. Xie F. Zhang L. Yan H. Huang J. (2022). Ferroptosis in cancer and cancer immunotherapy. Cancer Commun. (Lond) 42, 88–116. 10.1002/cac2.12250 35133083 PMC8822596

[B153] Zhao X. Jin Y. Li L. Xu L. Tang Z. Qi Y. (2019). MicroRNA-128-3p aggravates doxorubicin-induced liver injury by promoting oxidative stress via targeting Sirtuin-1. Pharmacol. Res. 146, 104276. 10.1016/j.phrs.2019.104276 31112750

[B154] Zhu J. Zhang X. Xie H. Wang Y. Zhang X. Lin Z. (2021). Cardiomyocyte Stim1 deficiency exacerbates doxorubicin cardiotoxicity by magnification of endoplasmic reticulum stress. J. Inflamm. Res. 14, 3945–3958. 10.2147/JIR.S304520 34421306 PMC8373307

[B155] Zoroddu M. A. Aaseth J. Crisponi G. Medici S. Peana M. Nurchi V. M. (2019). The essential metals for humans: a brief overview. J. Inorg. Biochem. 195, 120–129. 10.1016/j.jinorgbio.2019.03.013 30939379

